# The Regulatory Protein RosR Affects *Rhizobium leguminosarum* bv. *trifolii* Protein Profiles, Cell Surface Properties, and Symbiosis with Clover

**DOI:** 10.3389/fmicb.2016.01302

**Published:** 2016-08-23

**Authors:** Kamila Rachwał, Aleksandra Boguszewska, Joanna Kopcińska, Magdalena Karaś, Marek Tchórzewski, Monika Janczarek

**Affiliations:** ^1^Department of Genetics and Microbiology, Institute of Microbiology and Biotechnology, Maria Curie-Skłodowska UniversityLublin, Poland; ^2^Department of Molecular Biology, Institute of Microbiology and Biotechnology, Maria Curie-Skłodowska UniversityLublin, Poland; ^3^Department of Botany, Faculty of Agriculture and Biology, Warsaw University of Life SciencesWarsaw, Poland

**Keywords:** *rosR* gene, *Rhizobium leguminosarum*, extracellular proteins, membrane proteins, envelope properties, clover, symbiosis

## Abstract

*Rhizobium leguminosarum* bv. *trifolii* is capable of establishing a symbiotic relationship with plants from the genus *Trifolium*. Previously, a regulatory protein encoded by *rosR* was identified and characterized in this bacterium. RosR possesses a Cys_2_-His_2_-type zinc finger motif and belongs to Ros/MucR family of rhizobial transcriptional regulators. Transcriptome profiling of the *rosR* mutant revealed a role of this protein in several cellular processes, including the synthesis of cell-surface components and polysaccharides, motility, and bacterial metabolism. Here, we show that a mutation in *rosR* resulted in considerable changes in *R. leguminosarum* bv. *trifolii* protein profiles. Extracellular, membrane, and periplasmic protein profiles of *R. leguminosarum* bv. *trifolii* wild type and the *rosR* mutant were examined, and proteins with substantially different abundances between these strains were identified. Compared with the wild type, extracellular fraction of the *rosR* mutant contained greater amounts of several proteins, including Ca^2+^-binding cadherin-like proteins, a RTX-like protein, autoaggregation protein RapA1, and flagellins FlaA and FlaB. In contrast, several proteins involved in the uptake of various substrates were less abundant in the mutant strain (DppA, BraC, and SfuA). In addition, differences were observed in membrane proteins of the mutant and wild-type strains, which mainly concerned various transport system components. Using atomic force microscopy (AFM) imaging, we characterized the topography and surface properties of the *rosR* mutant and wild-type cells. We found that the mutation in *rosR* gene also affected surface properties of *R. leguminosarum* bv. *trifolii*. The mutant cells were significantly more hydrophobic than the wild-type cells, and their outer membrane was three times more permeable to the hydrophobic dye *N*-phenyl-1-naphthylamine. The mutation of *rosR* also caused defects in bacterial symbiotic interaction with clover plants. Compared with the wild type, the *rosR* mutant infected host plant roots much less effectively and its nodule occupation was disturbed. At the ultrastructural level, the most striking differences between the mutant and the wild-type nodules concerned the structure of infection threads, release of bacteria, and bacteroid differentiation. This confirms an essential role of RosR in establishment of successful symbiotic interaction of *R. leguminosarum* bv. *trifolii* with clover plants.

## Introduction

*Rhizobium leguminosarum* bv. *trifolii* is a soil bacterium that establishes a nitrogen-fixing symbiosis with clovers (*Trifolium* spp.). These plants belong to legumes (*Fabaceae*), a large and economically important group of plants represented by 18,000 species that are a source of food, feed, and biofuels, and can be used in many industrial applications (Olivares et al., 2013; Gresshoff et al., 2015). The distinctive ability of rhizobia to enter into a symbiosis with legumes leads to the formation of special organs, called nodules, on host plant roots (Janczarek et al., 2015b). Inside nodules, rhizobial cells differentiate into bacteroids that convert atmospheric dinitrogen to ammonia, a compound that can be used by the plant (Łotocka et al., 1997; Ferguson et al., 2010). Symbiosis plays an essential role in many ecosystems, annually yielding ~200 million tons of nitrogen, an amount similar to that introduced into the environment in the form of artificial nitrogen fertilizers (Graham and Vance, 2003).

The establishment of symbiosis between the host plant and its symbiotic partner is a complex, multi-stage process, involving exchange of many signals, the best characterized of which are plant-derived flavonoids and rhizobial lipochitooligosaccharides called Nod factors (Oldroyd and Downie, 2008; Hassan and Mathesius, 2012; Janczarek et al., 2015b). Development of a successful symbiosis also requires the presence of certain proteins, cell-surface components, and low molecular weight metabolites of both the macro- and the microsymbiont. Legumes synthesize proteins, such as Nod factor receptors, signal transduction cascade proteins, lectins, trifolins, and remorins (Janczarek et al., 2015b). In the case of rhizobia, surface polysaccharides, such as exopolysaccharide (EPS), lipopolysaccharide (LPS), capsular polysaccharide, glucomannan, cyclic β-glucans, and cellulose fibrils play a significant role in the symbiotic process (Downie, 2010; Margaret et al., 2012; Crespo-Rivas et al., 2015). Recently, the role of EPS as a signal molecule crucial for early stages of root infection has been confirmed, and a plant receptor responsible for its recognition has been identified (Kawaharada et al., 2015). This polysaccharide is especially important in rhizobial symbioses (e.g., *R. leguminosarum* bvs. *trifolii* and *viciae*, and *S. meliloti*) with legumes that form indeterminate-type nodules (e.g., clover, vetch, peas, alfalfa), where it is involved in the initiation and propagation of tubular structures called infection threads (ITs) (Cheng and Walker, 1998). However, some exceptions were found, e.g., *Sinorhizobium fredii* HH103 is not reliant on EPS for nodulation of *Glycyrrhiza uralensis* plants that also form indeterminate-type nodules (Margaret-Oliver et al., 2012). EPS is a major component of the IT matrix which protects the bacteria from plant defense responses (D'Haeze et al., 2004; Rodríguez-Navarro et al., 2014). The significance of EPS in the symbiosis has been established based on the observation that EPS-deficient mutant strains of *R. leguminosarum* bvs. *trifolii* and *viciae*, and *S. meliloti* induced non-nitrogen-fixing nodules on roots of compatible host plants (Ivashina et al., 1994; Cheng and Walker, 1998; Janczarek and Urbanik-Sypniewska, 2013).

The adhesion of rhizobia to roots and root hair tips, the targets of infection, constitutes a very important stage in the initiation of symbiosis. This process involves both bacterial surface polysaccharides and secreted proteins (Downie, 2010). The first step of rhizobial attachment is pH-dependent, as has been evidenced for *R. leguminosarum*. Under acidic conditions, a plant root hair tip lectin and bacterial glucomannan are engaged in this process, whereas basic conditions require an extracellular rhizobial protein called rhicadhesin (Laus et al., 2006; Williams et al., 2008). The second step of attachment, when rhizobia bind tightly to host roots, requires cellulose fibrils. These structures enable the bacteria to aggregate and form a biofilm on plant root hair tips (Laus et al., 2005; Williams et al., 2008).

Furthermore, type I protein secretion system PrsDE plays a significant role in the attachment of *R. leguminosarum* cells to host plant roots, as it transports several proteins involved in this process (cadherins—calcium-binding adherence proteins, *Rhizobium*-adhering proteins) (Krehenbrink and Downie, 2008; Abdian et al., 2012). This transport system is also essential for host plant infection, since it exports proteins of symbiotic significance, such as the calcium-binding nodulation-signaling protein NodO and glycanases PlyA, PlyB, and PlyC, which cleave nascent EPS (Economou et al., 1990; Sutton et al., 1994; Finnie et al., 1997; Zorreguieta et al., 2000; Russo et al., 2006).

In addition, cellulase CelC2, exported via a general export pathway, is engaged in the initial step of legume infection by *R. leguminosarum* (Robledo et al., 2008). In a curled root hair, which is a site of bacterial entry into the host plant, this enzyme is able to erode the noncrystalline cellulose present in the root hair cell wall, thereby allowing rhizobia to penetrate root tissues.

Furthermore, RosR appears to play an important role in a symbiotic interaction of *R. leguminosarum* bv. *trifolii* with clover (Janczarek and Skorupska, 2007). RosR is a 15.7-kDa protein containing a Cys_2_His_2_-type zinc-finger motif at its C-terminus, and belongs to the family of Ros/MucR transcriptional regulators involved in the regulation of EPS synthesis in various rhizobial species, such as *Rhizobium etli, Sinorhizobium meliloti*, and *Agrobacterium tumefaciens* (D'Souza-Ault et al., 1993; Keller et al., 1995; Bittinger et al., 1997). Previously, it has been established that RosR negatively regulates transcription of its own gene and positively regulates *pssA* and other *pss* genes encoding glycosyltransferases involved in EPS synthesis in *R. leguminosarum* (Janczarek and Skorupska, 2007; Rachwał et al., 2015). A strain harboring a mutation in *rosR* produced three times less EPS than a wild-type strain and showed some changes in the O-chain of LPS (Janczarek et al., 2010). Moreover, transcriptome analysis of this mutant revealed a significant role of RosR in the regulation of the expression of a large group of genes related to motility, synthesis of cell-surface components, and other cellular processes (Rachwał et al., 2015). A considerable majority of these genes were upregulated in the mutant, indicating that RosR functions mainly as a repressor.

The objective of the present study was to establish how a mutation in *rosR* gene influences protein levels in *R. leguminosarum* bv. *trifolii*, and whether subsequent changes in protein profiles alter mutant cell behavior in both free-living stage and during symbiosis with clover. Here, we studied the effect of *rosR* mutation on the profiles of extracellular, membrane, and periplasmic proteins of *R. leguminosarum* bv. *trifolii*, and identified proteins whose abundances were significantly different between the *rosR* mutant and the wild-type strain. Moreover, alterations in the cell surface topography and membrane integrity of these bacteria were detected by atomic force microscopy (AFM). In addition, the effects of *rosR* mutation on the symbiotic properties of *R. leguminosarum* bv. *trifolii* with clover plants were examined. Occupation of host root nodules was monitored using *gusA*-tagged bacteria and light microscopy, and the structure of *rosR* mutant nodules was characterized using electron microscopy. Our study underscores an essential role of RosR in establishment of successful symbiotic interaction of *R. leguminosarum* bv. *trifolii* with clover plants.

## Materials and methods

### Bacterial strains and growth conditions

The wild-type strain *R. leguminosarum* bv. *trifolii* Rt24.2 and its derivatives Rt2472, Rt2472(pRC24), and Rt2472(pBR1) had been described previously (Janczarek et al., 2009). The Rt2472 mutant obtained via random mutagenesis had a mini-Tn*5* transposon located inside the *rosR* coding region, while the Rt2472(pRC24) and Rt2472(pBR1) strains had the *rosR* mutation complemented by *rosR* introduced on pRC24 and pBR1 plasmids, respectively. All *R. leguminosarum* bv. *trifolii* strains were grown in tryptone-yeast extract medium (TY) (Beringer, 1974) at 28°C with agitation. Antibiotics were used at the following concentrations: rifampicin, 40 μg ml^−1^; kanamycin, 40 μg ml^−1^; and tetracycline, 10 μg ml^−1^.

### Preparation of protein fractions

For the analysis of extracellular, membrane and periplasmic proteins, the rhizobial strains were grown for 3 days to an OD_600_ of 0.7 in 400 ml of TY medium. Bacterial cells were pelleted from the cultures by centrifugation for 40 min at 10,000 × *g* at 4°C and then used for isolation of membrane proteins according to a method described previously (Janczarek et al., 2010). Culture supernatants after the second centrifugation were used to obtain extracellular proteins as described by Krehenbrink and Downie (2008). Briefly, 20% trichloroacetic acid (TCA) (w/v) was added to the culture supernatant to a final concentration of 7% (w/v), gently mixed and incubated for 3 h on ice. Then, the supernatant proteins were pelleted by centrifugation for 1 h at 15,000 × *g* at 4°C. The pellet was washed twice with 80% acetone (v/v) to remove residual TCA, air-dried and resuspended in SDS-PAGE sample buffer.

For the preparation of membrane proteins, bacterial pellets were washed in 50 mM Tris-HCl (pH 7.4), centrifuged for 30 min (10,000 × *g*, 4°C), and resuspended in 200 mM Tris-HCl (pH 8.0). Then, the cells were disrupted by sonication using the Misonix XL 2929 Sonicator Ultrasonic Processor (Misonix, Farmingdale, NY, U.S.A.). Unbroken cells were removed by centrifugation, and the supernatant was transferred on top of a two-step sucrose gradient in a centrifuge tube [top, 5 ml of 17% (w/v) sucrose; bottom, 1 ml of 55% (w/v) sucrose], and centrifuged at 30,000 × *g* for 90 min. Membrane fractions were collected, 20% TCA was added to a final concentration of 7%, and the samples were left overnight at 4°C. Precipitation of membrane proteins was done in the same manner as for culture supernatant proteins.

Periplasmic proteins were isolated from the bacterial pellets according to a method described by Krehenbrink et al. (2011). For each strain analyzed, three independent isolations of the individual protein fractions (extracellular, membrane, and periplasmic) were performed.

### Protein separation in 1D and 2D electrophoresis

Separation of rhizobial proteins was performed by both 1D and 2D electrophoresis. Standard 1D separations were done using 10 μg of the individual protein fractions per well and 10–20% Criterion Precast gels (Criterion TGX, Bio-Rad, Hercules, CA, U.S.A.) (Krehenbrink and Downie, 2008). 2D-PAGE was performed using Ready Strip IPG strips (11 cm, pH 4–7, nonlinear) and 10–20% Precast gels. Protein fractions precipitated with acetone were solubilized in 9 M urea, and protein concentration was determined using the Bradford dye (Bio-Rad, Hercules, CA, U.S.A.). Then, 100 μg of proteins was resuspended in 200 μl of a rehydration/sample buffer (9 M urea, 2% CHAPS, 40 mM Tris, 20 mM DTT, 0.5% IPG buffer), and the IPG strip was actively rehydrated with a sample overnight at room temperature. Then, isoelectric-focusing (IEF) was run at a maximum current setting of 50 μA/strip, according to the manufacturer's protocol. After IEF, the IPG strips were equilibrated 2 × 15 min in 10 ml of an equilibration buffer (6 M urea, 50 mM Tris pH 8.8, 30% glycerol, 2% SDS, and bromophenol blue in traces) containing 20 mM DTT, and 2 × 15 min in 10 ml of the same equilibration buffer containing 250 mg iodoacetamide. After this step, the strips were placed on top of the 10–20% Criterion precast gels. SDS-PAGE electrophoresis was performed at a constant voltage of 150 V, and protein visualization was carried out using Brilliant Blue G-colloidal dye (Sigma-Aldrich, St. Louis, MO, U.S.A.). The obtained gels were scanned using GS-800 densitometer and analyzed using Quantity One 1-D and PDQuest 2-D analysis software (Bio-Rad, Hercules, CA, U.S.A.). Three independent separations in both 1D and 2D were performed for each protein (extracellular, membrane, and periplasmic) fraction of the wild-type and *rosR* mutant strains.

### Western blotting

To establish the purity of the individual protein fractions obtained from the rhizobial strains, immunoblotting was performed using polyclonal rabbit antibodies against two proteins: a cytoplasmic protein PssB (Janczarek and Skorupska, 2001) and an outer membrane protein PssN (Marczak et al., 2006). Cytoplasmic protein fractions from Rt24.2 and Rt2472 were used as positive controls for the anti-PssB antibodies. Ten micrograms each of the protein fractions from these strains was loaded onto the gels, separated in 1D electrophoresis, and transferred onto a polyvinylidene difluoride membrane (Immobilon P, Millipore) in a Tris/glycine/methanol buffer according to the manufacturer's recommendations (Bio-Rad mini Trans-Blot, Hercules, CA, U.S.A.). Further steps of protein detection were performed as described earlier (Janczarek et al., 2010).

### Gel image analysis, sample preparation, and protein identification

Protein spots were identified in high-resolution digitalized 2D gel images and analyzed by PDQuest 2-D software (Bio-Rad, Hercules, CA, U.S.A.). After background subtraction, the ratios of mean normalized spot volumes were calculated, and values of corresponding spots in Rt2472 and Rt24.2 were compared. This analysis was performed independently for each individual protein fraction (extracellular, membrane, and periplasmic) of the strains, three gels for each strain and fraction were examined. Spots containing proteins whose amounts differed considerably between the Rt2472 and Rt24.2 fractions were excised (approx. 1.5 × 1.5 mm) from 2D gels and analyzed by liquid chromatography coupled with mass spectrometry in the Laboratory of Mass Spectrometry, Institute of Biochemistry and Biophysics, Polish Academy of Sciences (Warsaw, Poland). Samples were subjected to a standard procedure of tryptic digest, during which proteins were reduced with 100 mM DTT (30 min, 56°C), alkylated with 0.5 M iodoacetamide (45 min, room temperature) and digested overnight with 10 ng ul^−1^ trypsin (Promega, Madison, WI, U.S.A.) at 37°C. The peptide mixtures were concentrated and desalted on a RP-C18 pre-column (Waters, Milford, MA, U.S.A.), and then peptide separation was performed on a nano-Ultra Performance Liquid Chromatography (UPLC) RP-C18 column (BEH130 C18 column, 75 μm i.d., 250 mm long, Waters, Milford, MA, U.S.A.) of a nanoACQUITY UPLC system, using a 160 min gradient of 5–30% acetonitrile. The column outlet was directly coupled to the Electrospray ionization (ESI) ion source of the Orbitrap Velos type mass spectrometer (Thermo Fisher Scientific, Waltham, MA, U.S.A.), working in the regime of a data-dependent mass spectrometry (MS) to MS/MS switch with HCD-type peptide fragmentation. An electrospray voltage of 2 kV was used. Raw data files were pre-processed with Mascot Distiller software (version 2.4.2.0, Matrix Science, Boston, MA, U.S.A.). The obtained peptide masses and fragmentation spectra were matched to in-house *R. leguminosarum* protein database using the Mascot search engine (Mascot Daemon v. 2.4.0, Mascot Server v. 2.4.1, Matrix Science, Boston, MA, U.S.A.) Protein sequences for *R. leguminosarum* bv. *viciae* 3841 and *R. leguminosarum* bv. *trifolii* WSM1325 strains available at NCBI (accession nos. NC_008378.1-NC_008384.1, NC_012848, NC_012850, NC_0128452, NC_012853, NC_0128454, NC_0128458) and proteins of Rt24.2 strain previously obtained by us in the course of a whole genome shotgun sequencing (acc. no. MAMO00000000, BioProject: PRJNA224116) were used. This database contained 35,158 sequences. The search parameters were as follows: enzyme specificity, trypsin; protein mass, unrestricted; mass values, monoisotopic; maximum number of missed cleavages, one. Alkylation of cysteine by carbamidomethylation was set as a fixed modification. Oxidation of methionine, phosphorylation of serine, threonine, tyrosine and ubiquitination of lysine were set as variable modifications. The peptide and fragment ion mass tolerances were determined separately for the individual LC–MS/MS runs using a procedure based on two database searches, with an intermittent mass measurement error recalibration step using DatViewer software, developed in-house (http://proteom.ibb.waw.pl/mscan/). The expected value threshold of 0.05 was used for the analysis, which means that all peptide identifications had a <1 in 20 chance of being a random match.

### Atomic force microscopy

Bacterial samples for AFM were prepared according to a method described by Zdybicka-Barabas et al. (2011). Briefly, 12-h cultures of the Rt24.2, Rt2472 and Rt2472(pRC24) strains were diluted in a fresh portion of the medium to OD_600_ = 0.1. Then, 100 μl of each culture was centrifuged at 8000 × *g* for 10 min, and the bacterial pellets obtained were gently washed twice with 200 μl of sterile apyrogenic water. After a final centrifugation, the bacteria were suspended in 5 μl of water, loaded onto mica disks and allowed to dry overnight at 22°C. The rhizobial cell surface was imaged using a NanoScope V AFM (Veeco, Oyster Bay, NY, U.S.A.) (Analytical Laboratory, Faculty of Chemistry, Maria Curie-Sklodowska University, Lublin, Poland). All measurements, with the exception of the DMT modulus (5N m^−1^ TAP150A, Bruker, Billerica, MA, U.S.A.), were done in the “Peak Force QNM” operation mode using a silicon tip with a spring constant of 20 N m^−1^ (NSG30, NT-MDT, Russia). The following parameters were analyzed: (i) the height and peak force errors showing the topography of the examined strains, (ii) DMT (Derjaguin, Muller and Toporov) modulus, adhesion and deformation reflecting bacterial cell surface stiffness, (iii) adhesion forces between the cell surface and the tip. The data were analyzed with Nanoscope Analysis ver. 1.40 software (Veeco, Oyster Bay, NY, U.S.A.). The values of average root-mean-square (RMS) roughness were calculated using 40 fields sized 150 × 150 nm from 0.5 × 0.5 μm images of the entire cell surface of three individual bacteria, each from three different samples. A paired Student's *t*-test was used to assess differences in the cell size and other parameters between the wild-type and *rosR* mutant strains. The three-dimensional images and section profiles of the rhizobial cells were generated using WSxM 5.0 software (Nanotec, Spain; Horcas et al., 2007).

### *N*-phenyl-1-naphthylamine uptake assay

Outer membrane permeability of Rt24.2, Rt2472 and Rt2472(pRC24) was determined using the *N*-phenyl-1-naphthylamine (NPN) uptake assay (Vanderlinde and Yost, 2012). To this end, bacteria were scraped from TY agar plates and resuspended in 5 mM HEPES buffer (pH 7.2) to an optical density OD_600_ = 0.2. Hundred microliters portions of the bacterial suspensions were mixed with 95 μl of the HEPES buffer and 5 μl of 0.5 mM NPN solution in acetone. Two controls for each strain analyzed were performed; a first contained 100 μl of the HEPES buffer and 100 μl of the bacterial suspension, whereas a second contained 95 μl of the HEPES buffer and 5 μl of the NPN solution. The intensity of fluorescence was measured in the Tecan Infinite M1000 PRO microplate reader (Life Sciences, U.S.A.) for 15 min at 3-min intervals using excitation and emission wavelengths of 355 and 405 nm, respectively. To standardize the data, viable cells from the bacterial suspensions were counted in the plate assay. Data are reported as relative fluorescent units (RFU) per CFU and are means of two independent experiments with three biological repetitions for each strain analyzed.

### Cell hydrophobicity assay

To determine cell-surface hydrophobicity of the rhizobial strains, we used the two-phase method according to Neu and Poralla (1990) with minor modifications. Bacteria obtained from agar plates were suspended in PUM buffer (22.2 g K_2_HPO_4_ × H_2_O, 7.26 g KH_2_PO_4_, 1.8 g urea, 0.2 g MgSO_4_ × 7H_2_O, 1 l water) to an optical density of about 0.5 (OD_1_) at 405 nm. Hundred microliters of dodecane (Sigma-Aldrich, St. Louis, MO, U.S.A.) was added to 200-μl aliquots of the bacterial suspensions and left for 15 min at room temperature; this step was followed by exhaustive vortexing for 120 s. After 15-min equilibration, the optical density of the lower phase was measured (OD_a_) in a microplate reader (Biochrom Asys UVM 340, Biochrom, UK). The degree of hydrophobicity was calculated as follows: % hydrophobicity = 100 − 100(OD_a_/OD_1_). The experiment was performed twice with three biological repetitions for each strain analyzed.

### Plant nodulation assay

Seeds of red clover (*Trifolium pratense* cv. Diana) were surface-sterilized as described earlier (Janczarek et al., 2015a), placed on Fåhraeus agar plates (Vincent, 1970) and incubated for 2 days at 22°C. Then, seedlings were transferred to Fåhraeus slants and incubated for 4 days under natural light supplemented with artificial light (14 h at 24°C and 10 h at 18°C) in a greenhouse. After this time, the seedlings were inoculated with bacterial suspensions of OD_600_ = 0.1 (100-μl aliquot per plant), and their growth was continued for 42 days. Nodules appearing on clover roots were counted every week and the upper parts and roots of 6-week plants were measured and weighed. The experiment was repeated three times using 30 plants for each strain tested. In addition, the effect of *rosR* mutation on symbiotic proficiency of *R. leguminosarum* bv. *trifolii* was established in a 4-week experiment using Rt24.2, Rt2472, and Rt2472(pRC24) strains and *Trifolium repens* cv. Grassland Huia, and *Trifolium resupinatum* L., with *T. pratense* cv. Diana as a control host plant. Three biological replicates were performed for each strain, with 30 plants per replicate.

### Detection of β-glucuronidase activity and imaging of nodules

Rt24.2, Rt2472, and Rt2472(pBR1) cells inside clover root nodules were detected using these bacteria tagged with pJBA21Tc plasmid containing *gusA* for β-glucuronidase (Wielbo and Skorupska, 2001). β-glucuronidase activity in the rhizobial cells occupying clover nodules was detected by staining whole roots in 50 mM sodium phosphate buffer (pH 7.2) containing 50 μg ml^−1^ of 5-bromo-4-chloro-3-indolyl-β-D-glucuronide for 3 h (Janczarek et al., 2015a). The nodules were imaged under an OPTIPHOT2 light microscope equipped with a DS-Fil, 5 Megapixel color camera (Nikon Instruments, Melville, NY, U.S.A).

### Electron microscopy and H_2_O_2_ detection in nodules

A detailed structure of *rosR* mutant nodules was characterized using electron microscopy. The plant material was prepared for TEM analysis as described earlier (Janczarek et al., 2015a). The localization of H_2_O_2_ in nodules occupied by Rt2472 and Rt24.2 was determined as described previously (D'Haeze et al., 2004; Kopcińska, 2009). H_2_O_2_ was localized based on the reaction with CeCl_3_ (Sigma-Aldrich, St. Louis, MO, U.S.A.) forming Ce-perhydroxides, which appear as black precipitates when observed by TEM (Bestwick et al., 1997).

## Results

### *rosR* mutation affects *R. leguminosarum* bv. *trifolii* protein profiles

**Figure 1:**
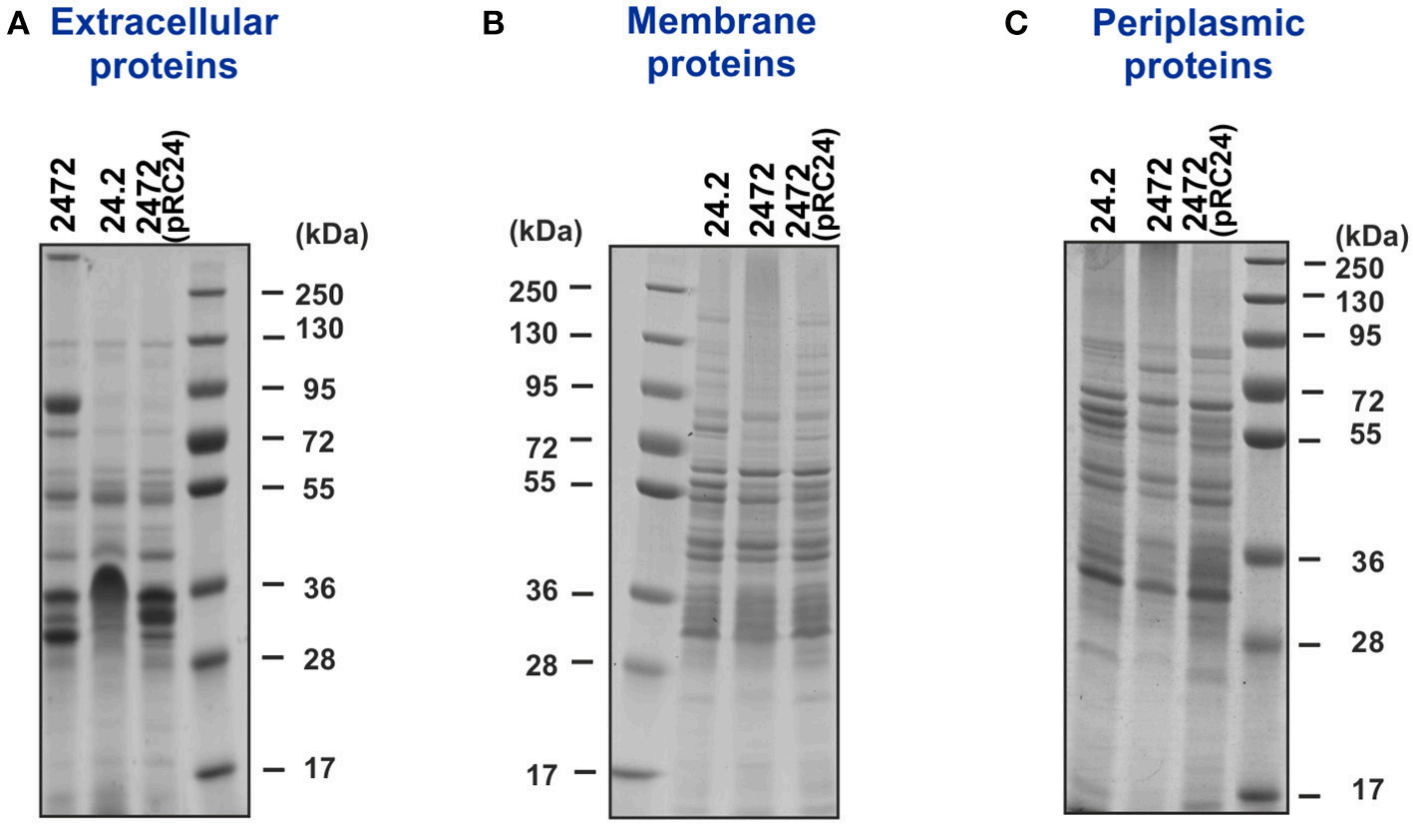
**Extracellular (A), membrane (B), and periplasmic (C) protein fractions of ***R. leguminosarum*** bv. ***trifolii*** Rt24.2, Rt2472, and Rt2472(pRC24) separated by 1D electrophoresis**. Proteins (10 μg) were loaded on each lane. Prestained protein ladder, 10–250 kDa (Thermo Scientific), was used as a molecular weight marker.

To establish whether the *rosR* mutation affected *R. leguminosarum* bv. *trifolii* protein profiles, extracellular, membrane, and periplasmic protein fractions were isolated from wild-type strain Rt24.2, Rt2472 *rosR* mutant, and Rt2472(pRC24) strain obtained by complementing the *rosR* mutation with pRC24 plasmid. The protein fractions were analyzed using 1D SDS-PAGE (Figures 1A–C). This revealed that *rosR* mutation affected protein profiles of *R. leguminosarum* bv. *trifolii*. The most significant alterations were observed in the extracellular (Figure 1A) and membrane (Figure 1B) protein fractions of the mutant in comparison with the wild-type strain. For the Rt2472(pRC24) strain, profiles of all the analyzed (extracellular, membrane, and periplasmic) protein fractions were identical to those of the wild-type strain, verifying that the defects associated with the *rosR* mutation were complemented by a plasmid-borne *rosR* gene.

**Figure 2:**
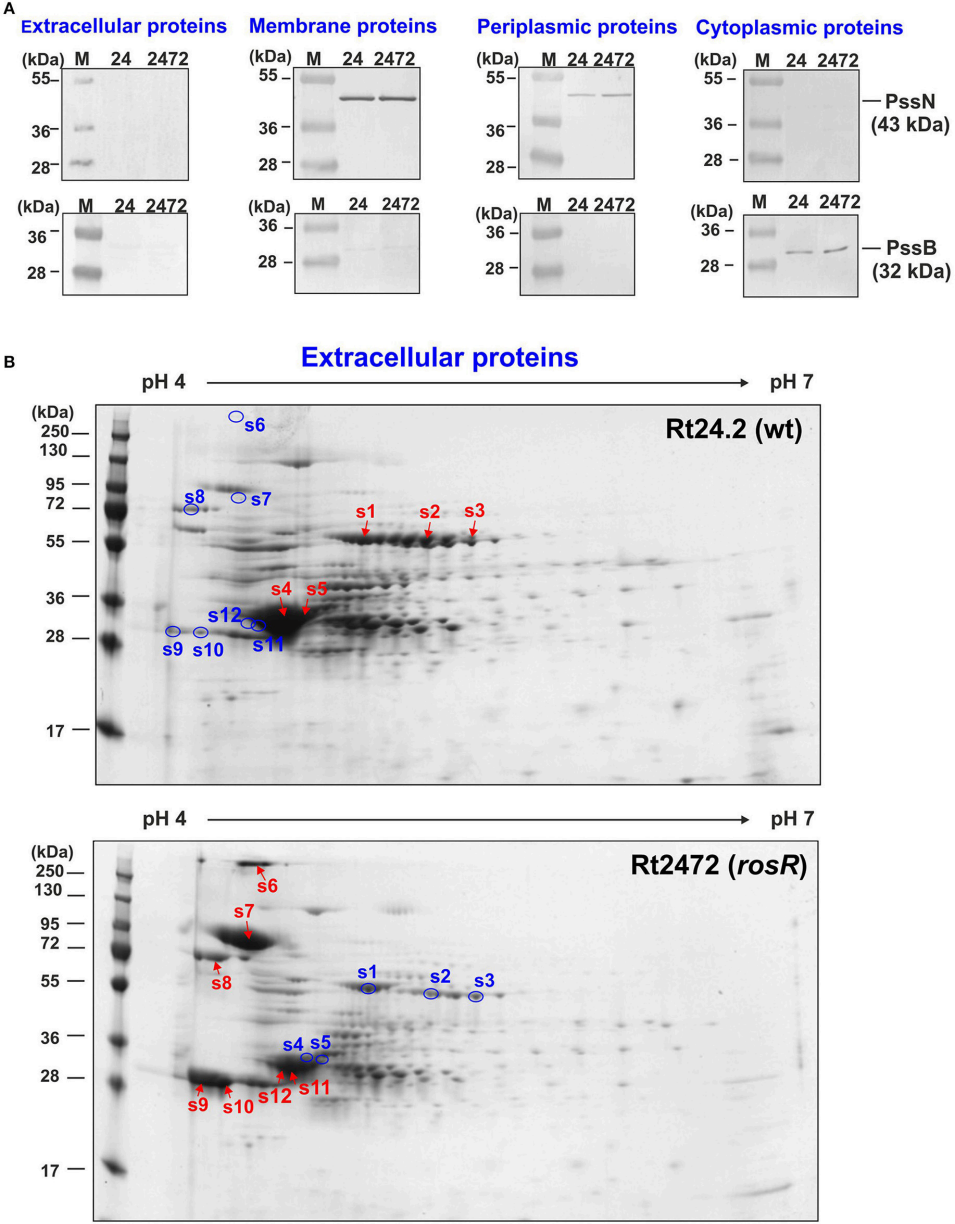
**Western blotting of extracellular, membrane, periplasmic, and cytoplasmic protein fractions of ***R. leguminosarum*** bv. ***trifolii*** Rt24.2 and Rt2472 strains (A)**. The assay was performed using polyclonal rabbit antibodies against 32-kDa cytoplasmic inositol monophosphatase PssB and 43-kDa outer membrane lipoprotein PssN with N-terminal domain directed to the periplasmic space. Ten micrograms of individual protein fractions was loaded on each lane. Abbreviations: 24- Rt24.2, 2472- Rt2472. **(B)** Two-dimensional electrophoretic profiles of extracellular proteins of Rt24.2 and Rt2472. Hundred micrograms of individual protein fractions were analyzed (molecular weight marker—prestained protein ladder, 10–250 kDa, Thermo Scientific). Spots containing proteins in higher amounts which were excised from the gel are marked by red arrows, whereas those containing diminished protein amounts were marked by blue circles.

To identify the proteins with quantitatively different abundances in the *rosR* mutant and wild-type strain, we performed 2D analyses of extracellular, membrane, and periplasmic protein fractions of those strains. Selected proteins were excised from gels and analyzed by mass spectrometry. The purity of the protein fractions was confirmed by western blotting with polyclonal rabbit antibodies against rhizobial cytoplasmic PssB and outer membrane PssN proteins (Figure 2A).

First, extracellular proteins of Rt2472 and Rt24.2 were analyzed by 2D PAGE. It was established that isoelectric points of rhizobial extracellular proteins fell within a narrow pH range, 4–6 (Figure 2B). Moreover, several of these proteins were present in smaller amounts in *rosR* mutant supernatant in comparison with that of the wild-type strain (spots 1–5) (Table 1). We identified some of those proteins as components of various transport systems. Among these were peptide-binding proteins DppA, branched-chain amino acid-binding protein BraC, and iron(III) ABC transporter substrate-binding protein SfuA. Fold-change ratios (Rt2472/Rt24.2) for these proteins were between 0.16 and 0.28. Interestingly, our data revealed that these types of proteins are present in the wild-type culture supernatant, similarly to findings for *R. leguminosarum* bv. *viciae* 3841 proteins homologous to Rt24.2 DppA and BraC described by Krehenbrink and Downie (2008).

**Table 1:** **Proteins of ***R. leguminosarum*** bv. ***trifolii*** Rt24.2 identified by mass spectrometry**.

**Spot no**.	**Accession**	**Identified protein^a^**	**Score**	**C (%)/F^b^**	**Theor. MW [kDa]/pI**	**Appar. MW [kDa]/pI**	**Protein fold change ratio^c^**	**Gene expression (Log2 fold change)^d^**
**EXTRACELLULAR PROTEINS**								
s1	OBY05349	Dipeptide ABC transporter, substrate-binding protein DppA (BAE36_20510)	3501	60/61	58.63/5.02	59/4.98	0.28 ± 0.02	−3.549 (Rt663_8)
s2	OBY08455	Dipeptide/oligopeptide-binding protein DppA-like (BAE36_04950)	878	27/13	58.25/5.93	58/5.42	0.24 ± 0.015	−3.643 (Rt619_161)
s3	OBY08455	Dipeptide/oligopeptide-binding protein DppA-like (BAE36_04950)	748	21/10	58.25/5.93	58/5.78	0.33 ± 0.02	−3.643 (Rt619_161)
s4	OBY07691	Amino acid ABC transporter, substrate-binding protein BraC (BAE36_07840)	1388	47/25	37.54/4.81	36/4.65	0.16 ± 0.01	−2.379 (Rt624_19)
s5	OBY03601	Iron(III) ABC transporter, substrate-binding protein SfuA (BAE36_30010)	1174	42/20	36.18/5.06	35/4.79	0.28 ± 0.02	−0.577 (Rt782_82)
s6	WP_018242663	Ca^2+^-binding cadherin-like protein containing VCBS domain	9533	39/106	69.60/4.02	250/4.3	18.5 ± 2.45	4.982 (Rt692_1)
s7	OBY08345	RTX toxin (BAE36_04365) homologous to Rleg_1548 of *R. leg*. WSM1325	32,404	66/504	69.38/4.41	75/4.29	12.33 ± 2.17	1.598 (Rt619_40)
s8	CAK10534	Ca^2+^-binding cadherin-like protein homologous to *R. leg*.3841 pRL100309	4897	14/112	60.74/4.08	70/4.12	3.12 ± 0.13	2.389 (Rt766_24)
s9	OBY04021	Autoaggregation protein RapA1 (BAE36_28000)	5053	72/61	24.35/4.20	28/4.15	4.08 ± 0.22	4.850 (Rt772_19)
s10	OBY04021	Autoaggregation protein RapA1 (BAE36_28000)	7922	72/86	24.35/4.20	28/4.21	4.57 ± 0.34	4.850 (Rt772_19)
s11	OBY06744	Flagellin FlaB (BAE36_13020)	12,959	46/174	31.22/4.51	31/4.55	11.31 ± 1.23	−4.601 (Rt634_13)
s12	OBY06743	Flagellin FlaA (BAE36_13015)	13,062	57/184	30.89/4.46	31/4.48	3.17 ± 0.25	−3.123 (Rt634_12)
**MEMBRANE PROTEINS**								
s13	OBY08371	Phasin family protein (BAE36_04495) similar to PhaP1 of *B. jap*. USDA110	7209	100/145	13.25/5.43	13/5.20	4.67 ± 0.23	0.331 (Rt619_66)
s14	OBY08371	Phasin family protein (BAE36_04495) similar to PhaP1 of *B. jap*. USDA110	6514	100/137	13.25/5.43	13/5.53	3.21 ± 0.19	0.331 (Rt619_66)
s15	OBY06791	Phasin (BAE36_13280) similar to PhaP3 of *B. japonicum* USDA110	4576	63/114	16.12/5.57	16/5.11	6.58 ± 0.57	0.163 (Rt634_68)
s16	OBY06791	Phasin (BAE36_13280) similar to PhaP3 of *B. japonicum* USDA110	9112	92/181	16.12/5.57	16/5.37	8.51 ± 0.75	0.163 (Rt634_68)
s17	OBY06791	Phasin (BAE36_13280) similar to PhaP3 of *B. japonicum* USDA110	8171	93/169	16.12/5.57	16/5.74	4.48 ± 0.33	0.163 (Rt634_68)
s18	OBY06743	Flagellin FlaA (BAE36_13015)	16,200	62/211	30.89/4.46	31/4.43	14.99 ± 1.41	−3.122 (Rt634_12)
s19	OBY08876	Urea/amides ABC transporter, ATP-binding protein UrtD (BAE36_02645)	592	25/9	27.29/5.72	27/5.75	0.17 ± 0.02	−1.837 (Rt616_146)
s20	OBY03633	Oxidoreductase (BAE36_30190)	10,424	91/192	30.55/5.93	32/6.15	0.22 ± 0.02	−1.402 (Rt782_118)
s21	OBY03144	Amidinotransferase (BAE36_32425)	3961	59/110	34.17/5.27	34/5.42	0.26 ± 0.015	−0.431 (Rt786_12)
s22	OBY05082	BMP family ABC transporter substrate-binding protein (BAE36_21985)	1516	49/27	38.71/6.17	40/6.18	0.21 ± 0.015	−1.545 (Rt683_3)
s23	OBY07384	FOF1 ATP synthase subunit beta (BAE36_10030)	18,157	93/310	50.94/5.02	52/5.09	0.33 ± 0.03	−0.936 (Rt627_73)
s24	OBY08480	Outer membrane protein assembly protein BamA (YaeT) (BAE36_04570)	27,116	70/424	84.61/4.83	85/4.78	0.17 ± 0.015	−1.853 (Rt619_81)
**PERIPLASMIC PROTEINS**								
s25	OBY06911	Catalase (KatG)/Hydroperoxidase HPI (BAE36_12380)	7550	52/142	79.61/5.46	73/5.36	0.11 ± 0.02	−0.396 (Rt632_41)
s26	OBY07688	peptide ABC transporter substrate-binding protein (BAE36_07825)	5740	54/121	59.15/5.52	58/5.28	0.32 ± 0.03	−1.756 (Rt624_16)

a *IDs of proteins identified in Rt24.2 draft genome sequence are given in parentheses (Acc. no. MAMO00000000)*.

b *C, sequence coverage; F, number of peptide fragments matched; R. leg., R. leguminosarum*.

c *Fold-change ratio Rt2472/Rt24.2 in protein abundance between Rt2472 and Rt24.2 strains. All proteins presented in this table showed statistically significant differences between the rosR mutant and the wild-type strain (P < 0.05, Student's t-test)*.

d *Differences in expression of genes encoding these proteins in Rt2472 and Rt24.2 are presented as log_2_ Rt2472/Rt24.2 fold change. The values are from Rachwał et al. (2015) (protein IDs used in that publication are given in parentheses)*.

On the other hand, a few proteins less abundant in the extracellular fraction of Rt24.2 were found in large quantities in the Rt2472 supernatant (spots 6–12). They included a RTX toxin-like protein, Ca^2+^-binding cadherin-like proteins, an autoaggregation protein RapA1, and flagellins FlaA and FlaB (Table 1). Among these proteins, the greatest differences in amounts between the *rosR* mutant and wild-type supernatants were observed for the cadherin-like protein in the spot 6 (18.5-fold change), RTX-like protein (12.33-fold change), FlaB (11.31-fold change), and RapA1 (4.57-fold change). RapA1 was previously characterized in *R. leguminosarum* as a protein with features similar to both rhicadhesins and bacterial lectins (Ausmees et al., 2001). Autoaggregation proteins (including RapA1) and cadherin-like proteins mediate cell adhesion in a calcium-dependent manner and are engaged in rhizobial attachment to root hairs (Williams et al., 2008). Also, two flagellins (FlaA and FlaB), components of flagella, located outside of bacterial cells, were present in significantly higher amounts in the mutant than in the wild-type supernatant.

**Figure 3:**
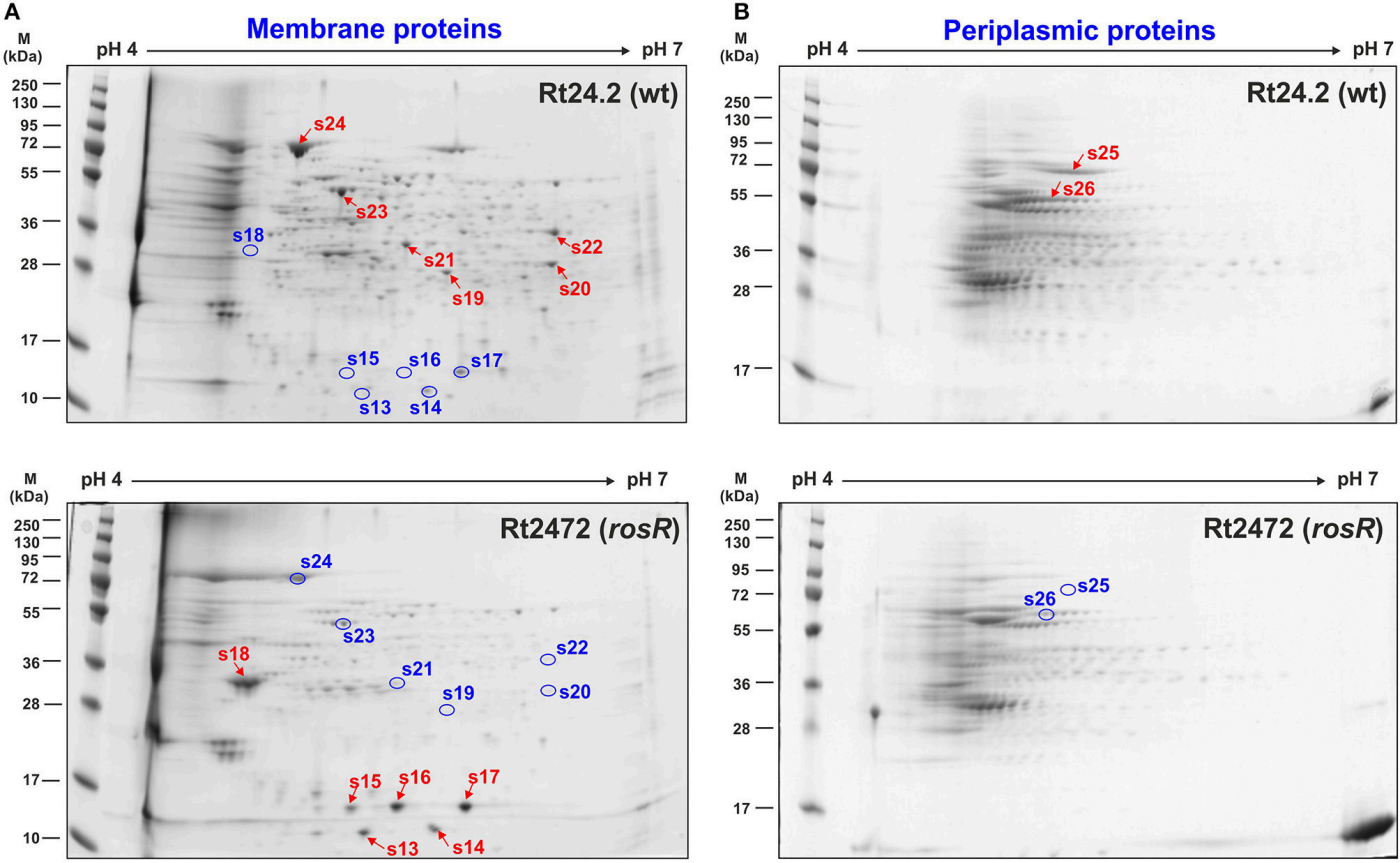
**Two-dimensional electrophoretic profiles of membrane (A) and periplasmic proteins (B) of Rt24.2 and Rt2472**. Individual protein fractions (100 μg) were analyzed (molecular weight marker, prestained protein ladder, 10–250 kDa, Thermo Scientific). Spots containing proteins in higher amounts which were excised from the gel are marked by red arrows, whereas those containing diminished protein amounts were marked by blue circles.

Similarly, differences were observed in the case of membrane protein profiles of Rt2472 and Rt24.2 (Figure 3A, Table 1). Among the proteins with levels significantly higher in the *rosR* mutant in relation to the wild-type strain were phasin-like proteins (spots 13–17) and flagellin FlaA (s.18). As has been reported for *Bradyrhizobium japonicum* USDA110, phasins play a major role in the accumulation and stabilization of poly-β-hydroxybutyrate, which is an intracellular carbon and energy storage polymer during free-living growth (Yoshida et al., 2013). This polymer is accumulated in the form of granules if a carbon source is provided in excess and if any other essential nutrient (e.g., nitrogen source) is limited. Proteins in spots 13–14 showed similarity (45.0% identity) to PhaP1 and those in spots 15–17 (28.8% identity) to PhaP3 proteins involved in granule stabilization in this bacterium.

In contrast, a few other proteins were less abundant in the *rosR* mutant in comparison with wild type (spots 19–24). Among these were components of various transport systems and enzymes: an ATP-binding protein UrtD of a urea/amide transport system, a BMP family ABC transporter substrate-binding protein, a FOF1 ATP synthase subunit β, an outer membrane protein assembly factor BamA, oxidoreductase, and amidinotransferase (Table 1). UrtD is a component of a transport system involved in uptake of urea/short-chain amides, whereas BAE36_10030 protein of Rt24.2 is a β-subunit of FOF1 synthase engaged in ATP synthesis. Protein BamA is a part of the outer membrane protein assembly complex involved in the assembly and insertion of β-barrel proteins into the outer membrane.

Next, a comparison of periplasmic protein fractions from Rt2472 and Rt24.2 was performed. The most conspicuous differences between these strains concerned two proteins, less pronounced in the *rosR* mutant profile in relation to that of the wild type: a protein identified as a catalase/hydroperoxidase HPI (KatG) (s.25) and a peptide-binding protein (s.26) (Figure 3B, Table 1). Their fold-change Rt2472/Rt24.2 ratios were 0.11 and 0.32, respectively. KatG is a bifunctional protein that protects bacteria from reactive oxygen species damage. As has been reported recently by Zhou et al. (2015), this protein plays an important role in the colonization of pea rhizosphere by *R. leguminosarum* bv. *viciae* 3841. The second identified protein, BAE36_07825, is a periplasmic component of an ABC-type transport system involved in the uptake of peptides.

Furthermore, a phenomenon known as “stuttering” was observed during 2D analysis of extracellular and periplasmic protein fractions (Figures 2B, 3B), and proteins in spots 2 and 3 were identified as the same dipeptide-binding protein, and those in spots 9 and 10 as RapA1. As determined by previous studies, this effect is observed in both bacterial and mammalian cells when they experience extreme starvation for certain amino acids (Parker et al., 1978; Rose, 1994; Holliday, 1995). This can lead to translational errors in individual proteins which, in a consequence, slightly change their isoelectric point. Since we used 72-h bacterial cultures for proteomics analyses, starvation for some amino acids could exist at this time of bacterial growth, resulting in “stuttering” of some rhizobial proteins.

To establish whether the differences observed in protein profiles of the *rosR* mutant in relation to the wild-type strain were caused by unspecific protein leakage from the cells or by altered expression of genes encoding these proteins, the proteomics data were compared with the previously obtained *rosR* mutant transcriptomic data (Rachwał et al., 2015). We found that proteins that were more abundant in the mutant profiles in relation to those of the wild type were encoded by genes that were also expressed at significantly higher levels in the mutant than in the wild-type background (log_2_ fold-change_Rt2472/Rt24.2_ values from 0.163 to 4.982) (Table 1). Importantly, the transcription of genes encoding proteins found in reduced amounts in Rt2472 in comparison with Rt24.2 was decreased in the *rosR* mutant (log_2_ fold-change_Rt2472/Rt24.2_ values from −0.396 to −4.601). These data indicate that the changes in protein profiles of the *rosR* mutant and the wild-type strain stem from differences in the expression levels of genes encoding these rhizobial proteins, and are associated with *rosR* mutation. Thus, proteomics results for an overwhelming majority of proteins presented in this study correlated well with transcriptomic data obtained for the *rosR* mutant. The only exceptions were flagellar proteins FlaA and FlaB detected in larger amounts in the extracellular and FlaA in the membrane fractions of the mutant than the wild-type strain. This may be associated with an impaired flagellation of the *rosR* mutant. Mutant cells produced only few and often very short flagella (Rachwał et al., 2015).

### *rosR* mutation affects the permeability, hydrophobicity, and topography of *R. leguminosarum* bv. *trifolii* cell surface

**Figure 4:**
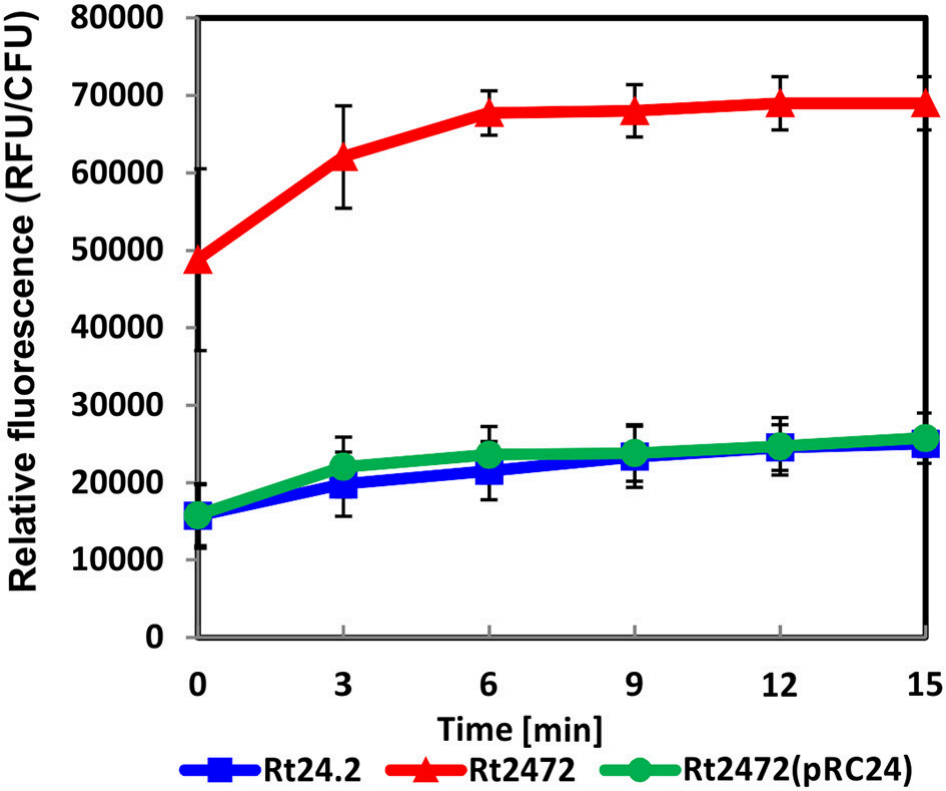
**Outer membrane permeability of ***R. leguminosarum*** bv. ***trifolii*** Rt24.2, Rt2472 and Rt2472(pRC24) strains using the ***N***-phenyl-1-naphthylamine uptake assay**. The experiment was repeated twice with three biological repetitions for each strain analyzed.

In addition to investigating the *rosR*-associated *R. leguminosarum* bv. *trifolii* protein profiles, we wanted to examine whether the *rosR* mutation affected the properties of rhizobial cell envelope. Membrane permeability of Rt24.2, Rt2472, and Rt2472(pRC24) were therefore determined using the NPN uptake assay. An intact bacterial outer membrane comprises a permeability barrier that prevents influx of hydrophobic substances, such as NPN. Once damaged, it allows the entry of NPN to the phospholipid layer, resulting in a prominent fluorescent signal. Using this approach, we established that both the wild-type Rt24.2 and complement Rt2472(pRC24) strains exhibited similarly low levels of fluorescence, calculated as relative values (RFU/CFU) (Figure 4). In contrast, *rosR* mutant cells exhibited a three-fold increase in fluorescence in comparison with Rt24.2 and Rt2472(pRC24), when assayed during a 15-min experiment. This indicated that the outer membrane of the *rosR* mutant was significantly more permeable than those of the wild-type and complemented *rosR* mutant strains.

Next, the hydrophobicity of Rt24.2, Rt2472, and Rt2472(pRC24) cells was determined using bacterial suspensions and dodecane. In this assay, the hydrophobicity values obtained for the parental strain and the complemented *rosR* mutant were similar [Rt24.2 = 15.89% ± 1.39 and Rt2472(pRC24) = 16.10% ± 2.84]. In contrast, *rosR* mutant cells were characterized by a nearly two-fold higher hydrophobicity than the control cells [Rt2472 = 30.06% ± 6.64; *P* < 0.01, Student's *t*-test].

**Figure 5:**
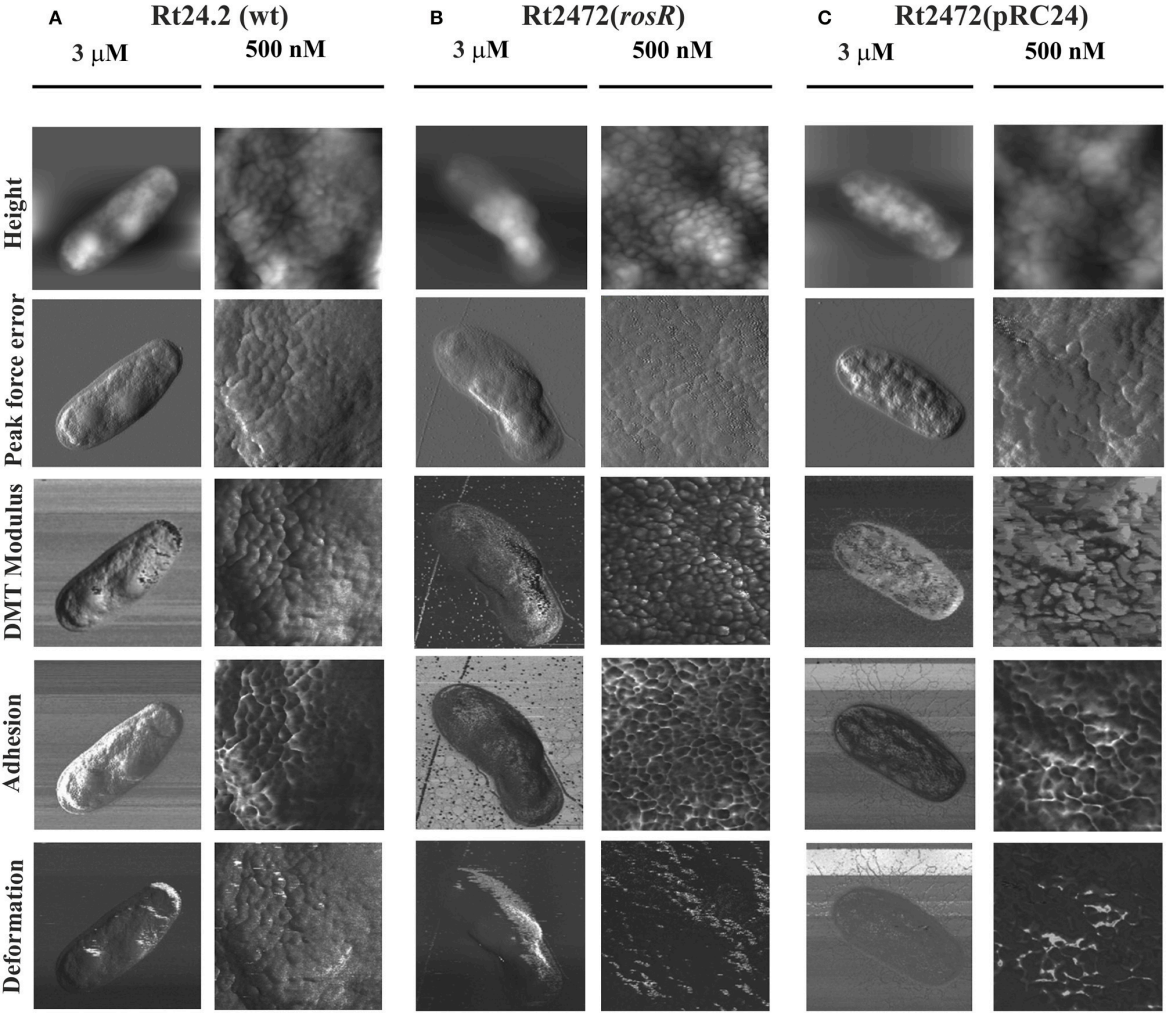
**AFM imaging of the wild-type ***R. leguminosarum*** bv. ***trifolii*** Rt24.2 (A), Rt2472 (B), and Rt2472(pRC24) (C) strains**. The height, peak force error, elasticity (DMT modulus), adhesion, and deformation images are presented. The brighter and darker image areas correspond to the higher and lower parameter values, respectively.

**Figure 6:**
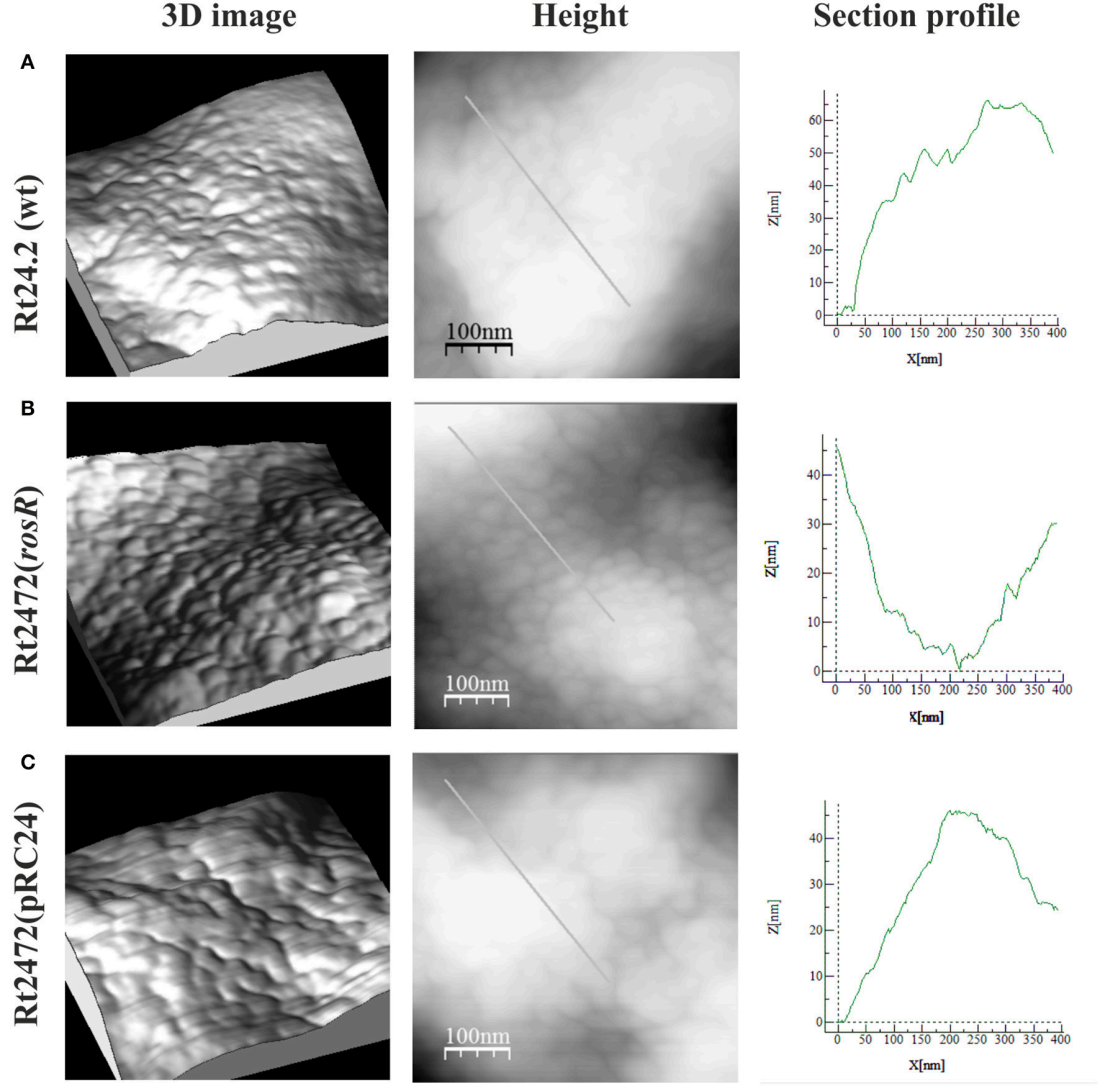
**AFM imaging of the wild-type ***R. leguminosarum*** bv. ***trifolii*** Rt24.2 (A), Rt2472 (B), and Rt2472(pRC24) (C) strains**. 3D images, height images, and section profiles corresponding to lines in the height images are presented.

To characterize the cell surface of the *rosR* mutant in more detail, AFM imaging of Rt24.2, Rt2472, and Rt2472(pRC24) cells was performed (Figures 5, 6). This experiment revealed differences in the topography and cell surface properties of the *rosR* mutant in relation to those of the control strains. Rt24.2 (Figures 5A, 6A) and Rt2472(pRC24) (Figures 5C, 6C) cells retained their normal rod shape with regularly spaced, small granules and irregular, long, and shallow grooves on the surface of their envelope. The average length and width of wild-type cells was 2.74 ± 0.064 and 0.879 ± 0.11 μm, respectively [Rt2472(pRC24) cells were 2.619 ± 0.272 μm long and 0.817 ± 0.178 μm wide]. In contrast, Rt2472 cells were shorter (2.191 ± 0.341 μm in length and 0.814 ± 0.089 μm in width) and had a more irregular shape, as seen in the height and peak force error images (Figure 5B). The surface of these cells was smoother and less granular than that of the parental cells, suggesting alterations in cell surface characteristics. Also, these cells, more frequently than the wild-type cells, had wide, but shallow, irregular depressions on their surface (Figure 6B). In addition, nanomechanical properties of the mutant were altered in comparison with the wild type, as shown in the peak force error, DMT modulus, adhesion and deformation images. Apart from these visually distinct properties, we also detected differences in calculated values corresponding to cell-surface RMS roughness, elasticity, and stiffness. The surface of the mutant cell was more inflexible than that of the wild-type cell, as reflected by a statistically significant 1.6-fold increase in DMT modulus [Rt2472 = 2.6 ± 0.41 GPa vs. Rt24.2 = 1.62 ± 0.34 GPa and Rt2472(pRC24) = 1.64 ± 0.15 GPa; *P* < 0.05, Student's *t*-test]. In addition, the roughness of the mutant cell surface was significantly lower in relation to that of the wild type [Rt2472 = 1.38 ± 0.18 nm, Rt24.2 = 2.38 ± 0.32 nm, Rt2472(pRC24) = 2.45 ± 0.55 nm; a difference statistically significant between the Rt2472 and Rt24.2, and between Rt2472 and Rt2472(pRC24), respectively; *P* < 0.05, Student's *t*-test]. These data indicate that the mutation in *rosR* caused changes in the topography and properties of the rhizobial cell envelope.

### *rosR* impacts *R. leguminosarum* bv. *trifolii* symbiosis with clover

**Figure 7:**
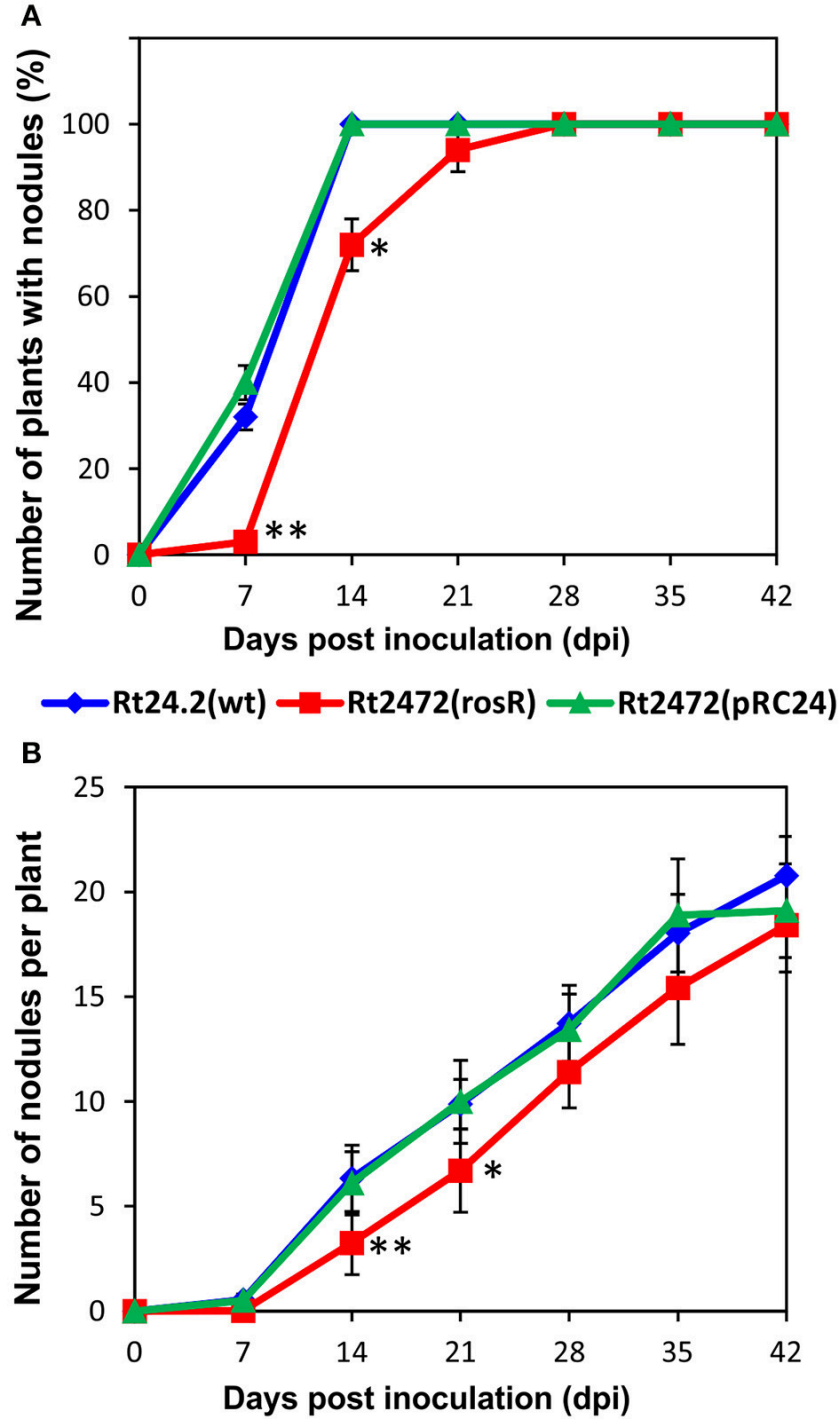
**The number of plants with nodules (A) and the number of nodules induced on clover (***T. pratense***) roots (B) by the wild-type ***R. leguminosarum*** bv. ***trifolii*** Rt24.2, Rt2472, and Rt2472(pRC24) strains**. Statistically significant differences between Rt2472 and Rt24.2, and Rt2472 and Rt2472(pRC24) are shown (^*^P < 0.05, ^**^P < 0.005; Student's *t*-test).

In further experiments, we investigated whether the distinct protein profiles and envelope properties of the *rosR* mutant affected its symbiotic relationship with the host plants. Red clover (*T. pratense*) seedlings were inoculated with Rt24.2, Rt2472, and Rt2472(pRC24), and grown for 6 weeks. This experiment verified that Rt24.2 and Rt2472(pRC24) were highly effective in infecting roots of this clover species. On day 7 post-inoculation (dpi), nodules were observed on 32%–40% of plants inoculated with these strains, and after next 7 days, all of the inoculated plants had nodules on their roots (Figure 7A). In contrast, the capacity of the *rosR* mutant to infect clover roots was significantly reduced. This was especially apparent during the first 14 days of the experiment. In addition, the total number of nodules induced by the *rosR* mutant was significantly lower than that induced by the wild-type strain (Figure 7B). After 6 weeks, clover plants inoculated with Rt2472 were small (Table 2), with yellow leaves, and developed white, often irregularly shaped nodules. Also, fresh shoot and root masses of plants inoculated with this mutant were much lower than those of plants infected by Rt24.2 and Rt2472(pRC24) strains. In contrast, the plants inoculated with Rt24.2 and Rt2472(pRC24) were tall and had green upper parts and many pink, functional nodules on their roots.

**Table 2:** **Symbiotic properties of ***R. leguminosarum*** bv. ***trifolii*** Rt24.2, ***rosR*** mutant Rt2472, and Rt2472 (pRC24) strains determined using ***T. pratense*** plants^a^**.

**Strain**	**Shoot length (mm)**	**Root length (mm)**	**Shoot fresh weight (mg)**	**Root fresh weight (mg)**
Rt24.2 (wt)	89.1 ± 6.1	114.3 ± 8.3	61.1 ± 7.0	57.3 ± 6.5
Rt2472 (*rosR*)	66.9 ± 2.6^*^	93.1 ± 4.1^*^	37.2 ± 5.1^*^	44.1 ± 3.0^*^
Rt2472 (pRC24)	102.1 ± 7.7	125.6 ± 3.8	66.9 ± 8.2	62.2 ± 7.8

a *Clover plants were grown for 6 weeks. Three biological replicates were performed for each strain, with 30 plants per individual experiment (in total, 90 plants per strain were tested)*.

* *Statistically significant differences between Rt2472 and Rt24.2, and between Rt2472 and Rt2472(pRC24) strains (P < 0.05, Student's t-test)*.

In addition, the ability of Rt24.2 to establish symbiosis with other clover species and the effect of *rosR* mutation on the symbiotic proficiency of this strain were studied using *T. repens* and *T. resupinatum*, with *T. pratense* as a control host plant. We determined that Rt24.2 and Rt2472(pRC24) induced nodule formation on roots of the three tested *Trifolium* species (Table 3). However, based on several symbiotic parameters (i.e., the number of plants with nodules, average number of nodules per plant, shoot mass), *T. pratense* proved to be the best host plant for these strains. In the case of *T. resupinatum* and *T. repens*, the number of plants with root nodules and the number of nodules formed were both lower compared with *T. pratense*. Because of this, the average shoot weights of *T. resupinatum* and *T. repens* plants inoculated with Rt24.2 and Rt2472(pRC24) were not as high as those of *T. pratense*, although they were higher than those of uninoculated plants. A nearly two-fold increase of plant shoot mass in relation to uninoculated plants was observed for *T. pratense*, indicating that Rt24.2 [and also Rt2472(pRC24)] established the most effective symbiosis with this host plant from the clover species tested. The effect of *rosR* mutation on symbiotic properties of Rt2472 was also determined using these clover species (Table 3). It was observed that the *rosR* mutant nodulated all three host plants but the number of induced nodules was significantly lower and their appearance on the roots was delayed in comparison with those of the wild-type strain. A similar negative effect of *rosR* mutation on the symbiotic proficiency of *R. leguminosarum* bv*. trifolii* was observed for all the tested clover plants.

**Table 3:** **Symbiotic properties of ***R. leguminosarum*** bv. ***trifolii*** Rt24.2 and its derivatives determined with different ***Trifolium*** spp. plants^a^**.

**Symbiotic parameter**	***T. pratense*** **cv. Diana**				***T. repens*** **cv. Grasslands Huia**				***T. resupinatum*** **L**.			
	**Rt24.2**	**Rt2472**	**Rt2472 (pRC24)**	**un**.	**Rt24.2**	**Rt2472**	**Rt2472 (pRC24)**	**un**.	**Rt24.2**	**Rt2472**	**Rt2472 (pRC24)**	**un**.
**PLANTS WITH NODULES (%)**												
7 dpi	36 ± 3	4.5 ± 0.5^*^	38 ± 3	0	13 ± 2	2.5 ± 0.5^*^	12 ± 2	0	29 ± 2	3 ± 1^*^	28 ± 2	0
14 dpi	100 ± 0	59 ± 4^*^	100 ± 0	0	49 ± 4	23 ± 4^*^	52 ± 5	0	66 ± 5	29 ± 3^*^	64 ± 5	0
21 dpi	100 ± 0	78 ± 5^*^	100 ± 0	0	59 ± 5	51 ± 4^*^	62 ± 6	0	80 ± 5	42 ± 4^*^	81 ± 6	0
24 dpi	100 ± 0	89 ± 7^*^	100 ± 0	0	66 ± 7	58 ± 6^*^	69 ± 7	0	89 ± 6	51 ± 4^*^	87 ± 7	0
**NODULE NO PER PLANT**												
7 dpi	0.6 ± 0.2	0.1 ± 0^*^	0.7 ± 0.2	0	0.2 ± 0.1	0.1 ± 0	0.2 ± 0.1	0	0.5 ± 0.1	0.1 ± 0^*^	0.6 ± 0.1	0
14 dpi	3.6 ± 0.5	2.1 ± 0.2^*^	3.9 ± 0.6	0	1.1 ± 0.2	0.7 ± 0.2^*^	1.2 ± 0.2	0	2.5 ± 0.2	1.1 ± 0.1^*^	2.7 ± 0.3	0
21 dpi	6.9 ± 0.7	3.9 ± 0.4^*^	7.1 ± 0.8	0	2.2 ± 0.3	1.8 ± 0.3^*^	2.5 ± 0.4	0	4.4 ± 0.3	2.3 ± 0.3^*^	4.7 ± 0.4	0
24 dpi	8.7 ± 1.9	6.5 ± 1.3	8.9 ± 0.9	0	3.9 ± 0.5	2.2 ± 0.4^*^	3.8 ± 0.5	0	6.5 ± 0.6	4.1 ± 0.5^*^	6.1 ± 0.5	0
Shoot length (mm)	81.1 ± 8.2	68.2 ± 6.1	77.6 ± 8.3	61.4 ± 7.5	39.1 ± 5.0	34.6 ± 3.8	37.3 ± 4.8	32.1 ± 4.0	51.1 ± 6.4	39.1 ± 4.5^*^	50.8 ± 4.2	31.1 ± 3.3
Root length (mm)	66.2 ± 6.6	57.1 ± 6.7	64.4 ± 6.3	54.4 ± 4.7	33.2 ± 4.8	30.3 ± 4.3	34.2 ± 4.7	28.2 ± 3.7	45.2 ± 4.3	40.4 ± 4.2	48.2 ± 5.1	37.2 ± 3.2
Shoot fresh weight (mg)	51.3 ± 4.6	30.1 ± 4.7^*^	52.4 ± 5.5	28.3 ± 3.4	12.0 ± 2.2	8.1 ± 2.5^*^	11.2 ± 3.1	7.2 ± 3.4	23.2 ± 3.4	15.1 ± 2.0^*^	22.2 ± 3.1	13.1 ± 2.0
Root fresh weight (mg)	28.1 ± 3.3	18.2 ± 2.5^*^	29.3 ± 4.8	14.4 ± 2.7	6.1 ± 3.5	4.5 ± 2.0	5.2 ± 3.1	4.1 ± 2.0	11.1 ± 2.1	9.0 ± 2.1	12.0 ± 2.3	8.1 ± 2.2

a *Clover plants were grown for 4 weeks. The experiment was performed in triplicate using 30 plants per each strain tested in each experiment. Data presented are averaged means ± SD. un., uninoculated plants; dpi, days post inoculation*.

* *Show statistically significant differences between Rt2472 and Rt24.2, and between Rt2472 and Rt2472(pRC24) strains determined for each Trifolium species (P < 0.05, Student's t-test)*.

**Figure 8:**
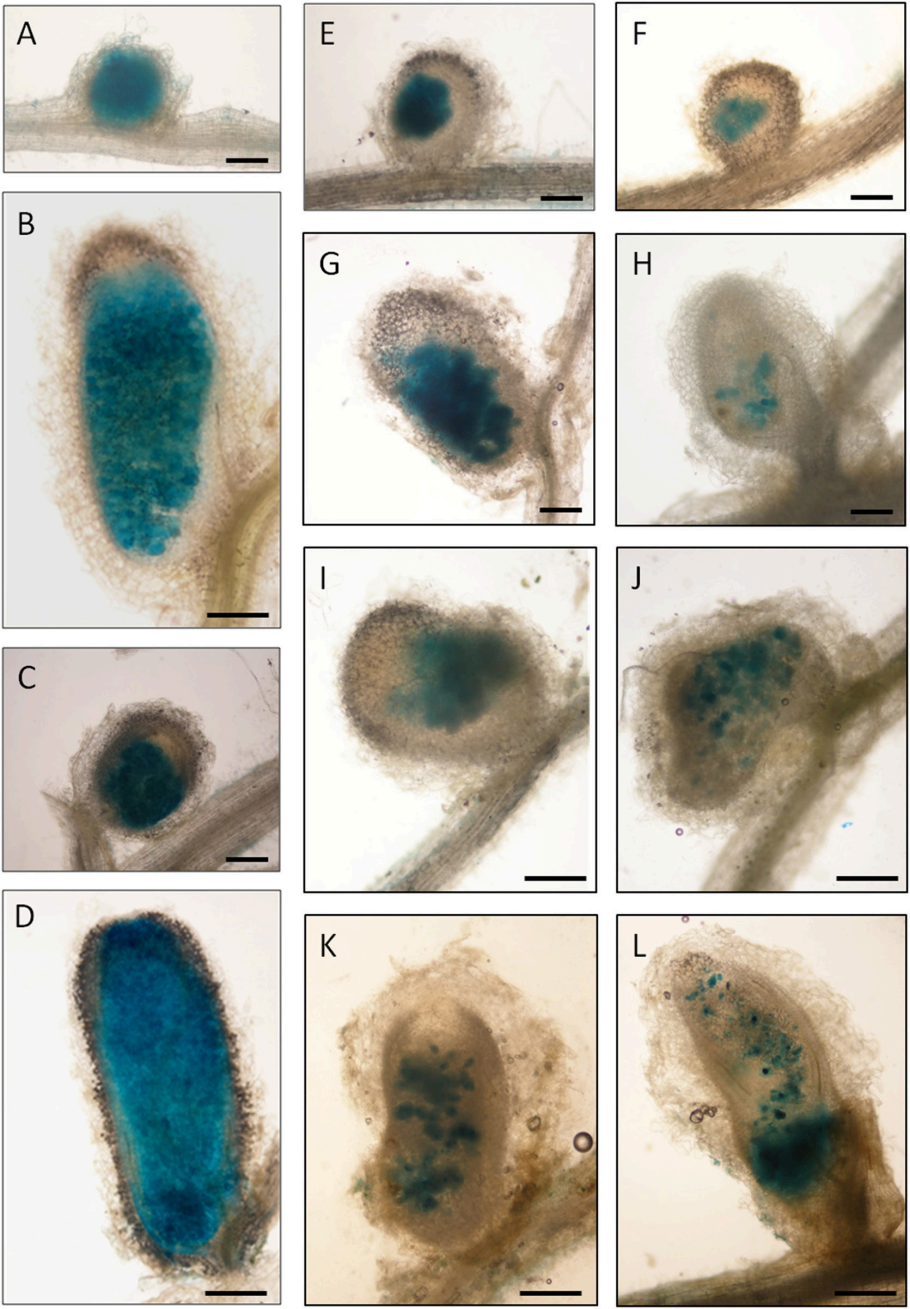
**Light microscopy of clover (***T. pratense***) root nodules induced by the ***R. leguminosarum*** bv. ***trifolii*** strain Rt24.2, Rt2472 ***rosR*** mutant and Rt2472(pBR1) strains harboring pJBA21Tc plasmid with ***gusA*** reporter gene for β-glucuronidase. (A,B)** Rt24.2 nodules at 7 and 28 days post inoculation (dpi), respectively; **(C,D)** Rt2472(pBR1) nodules at 7 and 28 dpi, respectively; **(E–L)** nodules occupied by Rt2472: **(E,F)** 7-dpi nodules, **(G–I)** 14-dpi nodules, **(J)** a 21-dpi nodule, **(K)** a 24-dpi nodule, and **(L)** a 30-dpi nodule, respectively. Bar = 150 μm **(A,C,E–H)**; bar = 300 μm **(B,D,I–L)**.

To compare the occupation of nodules by the *rosR* mutant and wild-type strain, Rt24.2, Rt2472, and Rt2472(pBR1) strains tagged with the pJBA21Tc plasmid, containing *gusA* for β-glucuronidase, and *T. pratense* as the host plant, were used. We observed that Rt24.2 and Rt2472(pBR1) occupied the nodules very effectively and these bacteria were found in both young (7-dpi; Figures 8A,C) and older (28-dpi) nodules (Figures 8B,D). In 28-dpi nodules, β-glucuronidase activity was detected in all zones, with the exception of the meristem (i.e., infection zone II, interzone II–III, nitrogen-fixing zone III, and senescent zone IV). In contrast, Rt2472 occupied the nodules much less effectively than the control strains. Although this mutant was detected inside the majority of young nodules (Figures 8E,F), some of the nodules were not occupied by this bacterium (data not shown). Rt2472 was unevenly distributed in the zones of older nodules (14-, 21-, and 24-dpi; Figures 8G–K), in contrast with wild-type nodules. In 30-dpi nodules, β-glucuronidase activity was detected only in a small number of nodule cells, a majority of which were located in the senescence zone (Figure 8L). This observation suggested premature degeneration and death of the *rosR* mutant inside host plant cells.

### *rosR* mutation affects the structure of clover root nodules and H_2_O_2_ deposition

**Figure 9:**
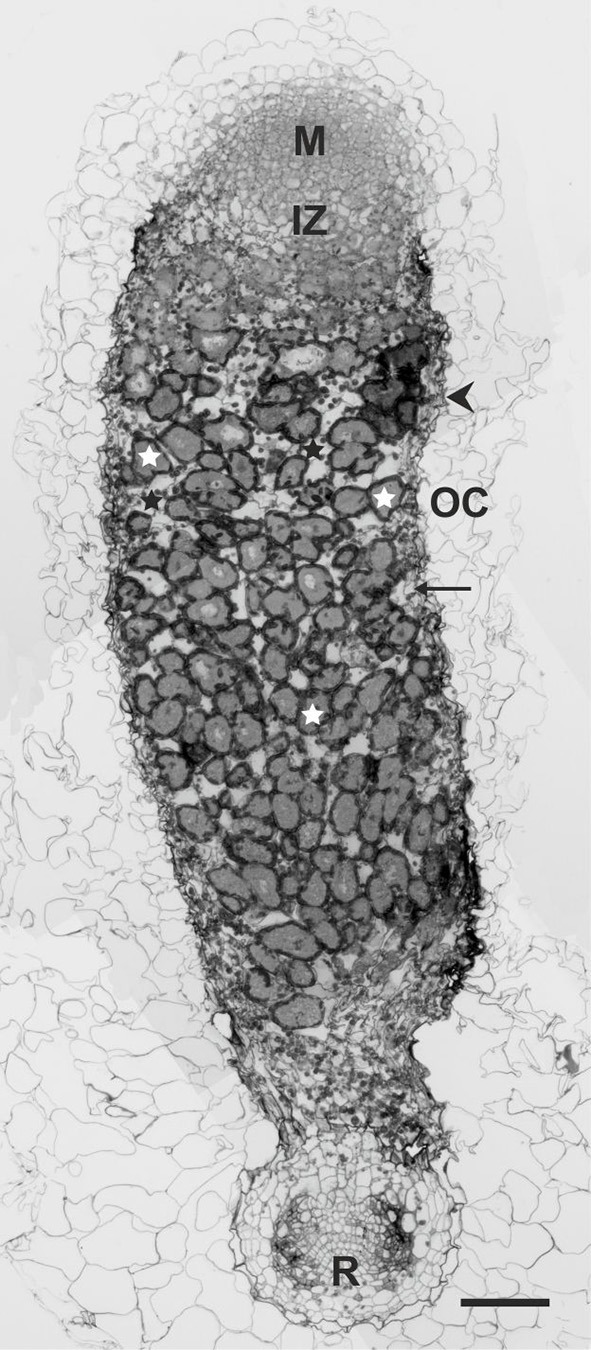
**A semi-thin section of a 21-dpi ***T. pratense*** root nodule induced by the ***R. leguminosarum*** bv. ***trifolii rosR*** mutant Rt2472**. Abbreviations: M, meristematic zone; IZ, infection zone; OC, outer cortex; R, root; arrow, inner cortex; arrow head, nodule endodermis; white asterisk, infected cells; black asterisk, uninfected cells. Bar = 100 μm.

Finally, we undertook a detailed analysis of the structure of nodules induced by the *rosR* mutant on clover roots. Previously, we have characterized nodules induced by the parental strain Rt24.2 on roots of this plant, which exhibited a typical structure with all zones present, including a large nitrogen-fixation zone (Janczarek et al., 2015a). The infection threads had normal thread walls and large amounts of thread matrix, and properly differentiated bacteroids were found inside mature infected plant cells. The anatomy of nodules induced by Rt2472 resembled that of the wild-type nodules, i.e., with bacteroidal tissue surrounded by two to three layers of inner cortex with vascular bundles, a single layer of endodermis, and the outer cortex comprised by large, loosely arranged cells. Nevertheless, mutant nodule development was disturbed. In a 21-dpi nodule induced by Rt2472, a bacteria-free meristem zone I and an infection zone II were distinguished (Figure 9). However, no distinct interzone II–III and no nitrogen-fixing zone III were observed. A large part of the nodule contained cells with bacteria, including amyloplasts with very large starch grains and symbiosomes at various stages of degradation. This zone corresponded to a senescent zone IV in wild-type nodules.

**Figure 10:**
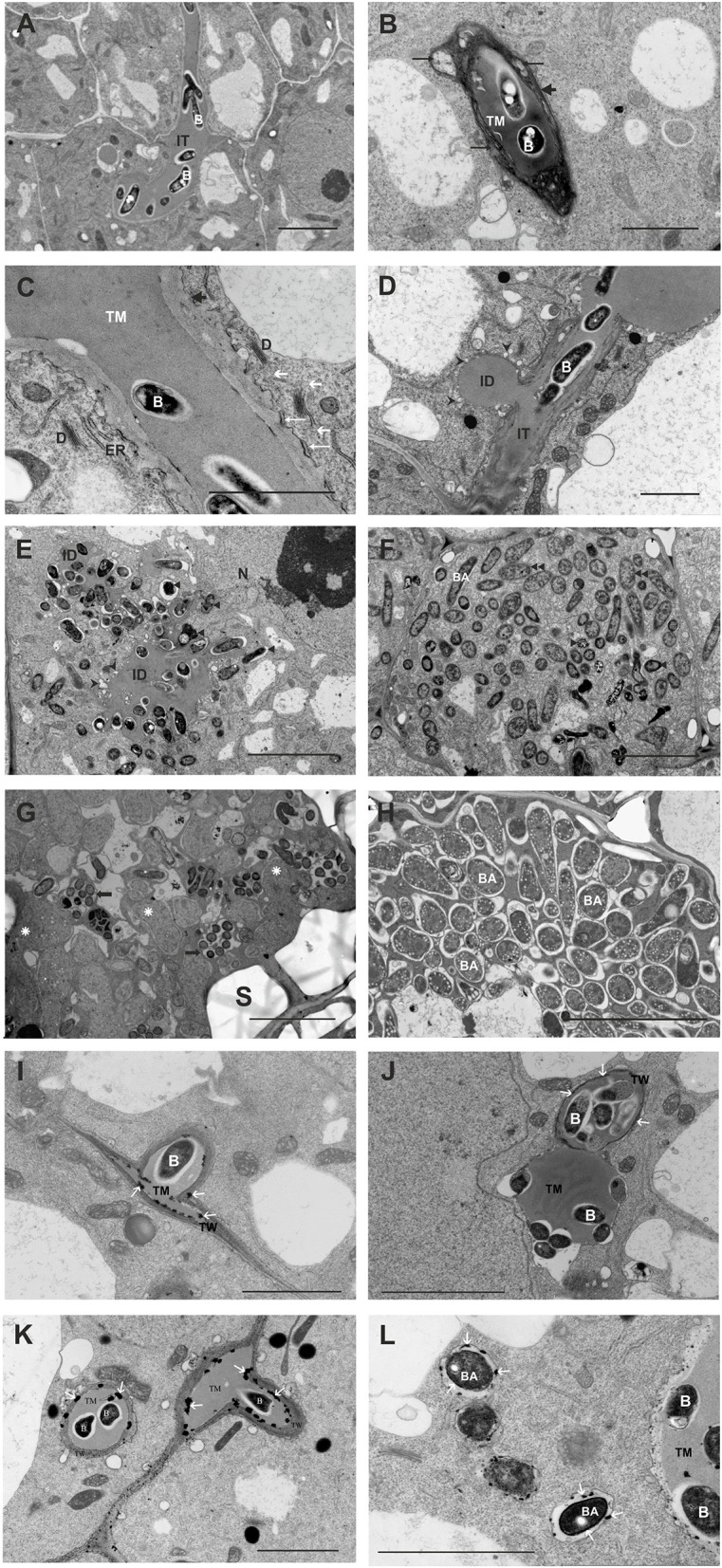
**Ultrastructure of wild-type (A) and ***rosR*** mutant infection threads (B,C)**. Abbreviations: IT, infection thread; TM, thread matrix; B, bacteria; D, dictyosome; ER, endoplasmic reticulum; short black arrow, thread wall; long black arrows, deposits of more translucent material between osmophilic layers of the thread wall; white long arrows, short ER cisterns in close contact with the thread membrane; short white arrows, transport vesicles. Bar = 5 μm. **(D,E)** Infection droplets containing *rosR* mutant bacteria. **(D)** A lateral infection droplet on an infection thread. **(E)** A large infection droplet inside the host cell. B, bacteria; ID, droplet; IT infection thread; N, nucleus; black triangle, degenerated bacteroids; black arrow heads, vesicles separated from the thread membrane surrounding the infection droplet. Bar = 2 μm for **(D)** and 5 μm for **(E)**. **(F)** Symbiosomes containing *rosR* mutant bacteroids in the young infected host cell; note three types of bacteroids present: BA, bacteroids similar to those observed in the early symbiosis zone of wild-type nodules; double triangle, differentiating bacteroids; triangle, degenerated bacteroids. Bar = 5 μm. **(G)** A mature infected host cell: S, starch grain; rosette, abnormally differentiated *rosR* mutant bacteroids; black arrows, lytic compartments with degrading bacteroids. Bar = 5 μm. **(H)** Symbiosomes containing wild-type bacteroids in a mature infected host cell: BA, normal bacteroids. Bar = 5 μm. H_2_O_2_ detection in Rt24.2 and Rt2472 nodules as electron-dense precipitates formed in the presence of cerium chloride **(I–L)**. H_2_O_2_ localization in the cell wall of a wild-type infection thread **(I,J)**. H_2_O_2_ precipitates (white arrows) present in the cell wall of the infection threads containing *rosR* mutant bacteria and on the surface of bacteroids and their peribacteroidal membranes **(K,L)**. Bar = 2.5 μm (for **I,J**), Bar = 5 μm (for **K,L**).

At the ultrastructural level, the most striking differences between the mutant and the wild-type nodules concerned the IT structure, bacteria release, and bacteroid differentiation (Figure 10). In contrast to the wild-type ITs (Figure 10A), mutant-induced ITs were wide, often branched, and surrounded by a thick wall (Figure 10B) that was not as uniformly fine-fibrillar as in wild-type ITs. This wall was significantly expanded and contained discontinuous layers of strongly osmophilic material between which a more homogenous and less osmophilic material was deposited (Figure 10B). Golgi bodies, rough ER, and numerous vesicles were present in the plant cell cytoplasm along the ITs. The vesicles were commonly found in the vicinity of Golgi bodies and in association with the IT membrane. Also, close contact between the short ER cisterns and the IT membrane was apparent. Bacteria within the IT were surrounded by a thread matrix (Figure 10C). Moreover, the release of the *rosR* mutant cells from the ITs was disturbed. In contrast to the wild-type ITs, the tips of mutant ITs were usually swollen, had knobs and protrusions, and frequently occupied a considerable part of the host cell cytoplasm. In addition, many small and “empty” vesicles were formed by the plasma membrane surrounding the IT tips. Sometimes, lateral, un-walled bulges of ITs were observed (Figure 10D), not present in the wild-type ITs. The lateral bulges are hypothesized to be initial infection droplets mediating the release of bacteria into the cytoplasm (Newcomb, 1976). Simultaneous endocytosis of many bacterial cells was observed (Figure 10E). Bacteroid differentiation was also impaired in the *rosR* mutant. After release, the bacteria were enclosed in the peribacteroid membrane and differentiated into bacteroids. However, many of these released bacteria and recently formed bacteroids showed signs of premature intensive degeneration (electron-dense cytoplasm, irregular shape, locally enlarged periplasmic space, and membranous structures in the cytoplasm; Figures 10E,F). The significant impairment in bacteroid differentiation was observed as a fast, large-scale deformation of the cells (they were enlarged, swollen, abnormally shaped, had homogenous cytoplasm, and underwent rapid degradation; Figure 10G). Groups of small degenerated bacteroids inside numerous vacuole-like structures were observed. As a result of these disturbances, the nodules occupied by the *rosR* mutant were not effective in nitrogen fixation. This was in contrast with the normal, typically differentiated wild-type bacteroids found in mature infected nodule cells (Figure 10H).

Since H_2_O_2_ plays an important role in IT formation and elongation as well as plant defense responses, we also examined the accumulation of this compound inside clover root nodules containing Rt24.2 and Rt2472. When plants were inoculated with Rt24.2, H_2_O_2_ was present in the ITs in the infection zone of the nodule, principally between the cell wall and the matrix (Figures 10I,J). The most essential difference observed for *rosR* mutant nodules and wild-type nodules was that in the former, H_2_O_2_ also accumulated outside the IT wall (Figure 10K), in the peribacteroidal symbiosome membrane and on the surface of the contained bacteroids (Figure 10L). This suggests that the *rosR* mutant–clover interaction results in the production of higher amounts of H_2_O_2_ or a decrease in the effectiveness of elimination of this compound.

In conclusion, all these data indicate that Rt2472 strain symbiosis with clover is strongly impaired and that the impairment mainly involves the release of bacteria from ITs, bacteroid differentiation, and premature bacteroid senescence, which, in consequence, renders the nodules unable to fix nitrogen.

## Discussion

In this work, we demonstrate that *rosR* mutation affects protein profiles of *R. leguminosarum* bv. *trifolii* and the observed changes in protein content of the mutant resulted in alteration of its cell-surface properties and disturbances in symbiosis with clover plants. We found that the extracellular protein profile of the *rosR* mutant significantly differed from the wild type (Figures 1, 2). Several proteins were more abundant in the mutant fraction. These include: two Ca^2+^-binding cadherin-like proteins, RTX protein, autoaggregation protein RapA1, and flagellins FlaA and FlaB. On the other hand, abundances of a few proteins were strongly decreased in the mutant. Among them were dipeptide/oligopeptide-binding proteins DppA, branched-chain amino acid-binding protein BraC, and iron(III)-binding protein SfuA.

Another observation made herein was that membrane and periplasmic fraction proteins were altered in the *rosR* mutant in comparison with wild type (Figure 3). The most significant changes concerned elevated levels of flagellin FlaA and phasins PhaP1 and PhaP3 related to polyhydroxyalkanoate granules stabilization, and reduced levels of several transport system components (e.g., ATP-binding protein UrtD of the urea/amide transport system and BMP family substrate-binding protein) and membrane-associated proteins (e.g., outer membrane protein assembly factor BamA and FOF1 ATP synthase subunit β). In addition, bifunctional catalase/hydroperoxidase HPI (KatG) and a substrate-binding protein of peptide transport system were present in smaller amounts in the Rt2472 periplasmic space.

To establish whether the observed differences in protein abundances between the *rosR* mutant and the wild-type strain are associated with an unspecific protein leakage or changes in gene expression, we compared our proteomics results with transcriptomic data for these strains (Rachwał et al., 2015). A correlation with comparative transcriptome analysis data for genes encoding these proteins was found for a great majority of proteins identified in this study (the only exception concerned flagellins FlaA and FlaB) (Table 1). This suggests that the main cause for the observed alterations in protein amounts between the *rosR* mutant and wild-type strains was difference in gene expression in these strains. RNA-Seq analysis identified a large group of genes whose expression was regulated by RosR in *R. leguminosarum* bv. *trifolii* (Rachwał et al., 2015). Among these were genes related to transport and metabolism of various carbon and nitrogen sources and bacterial motility, as well as those involved in the synthesis of cell-surface polysaccharides (e.g., EPS, glucomannan, and gel-forming polysaccharide) and other components (e.g., RapA1 and PrsD). Our results indicate that wild-type levels of proteins whose abundances were changed in the *rosR* mutant are required for optimal bacterial cell-surface properties and successful symbiosis of *R. leguminosarum* bv. *trifolii* with clover. Secreted proteins, such as cadherin-like proteins and autoaggregation proteins (RapA1, RapA2, RapC), are important in the early stages of symbiosis, i.e., adhesion to and infection of host plant roots (Fauvart and Michiels, 2008; Mongiardini et al., 2008). Hence, significantly higher amounts of these proteins in the *rosR* mutant explain its considerably increased aggregation and formation of large clumps during both liquid culture and agar plate growth (Rachwał et al., 2015). These proteins are secreted by type I protein secretion system PrsDE (Ausmees et al., 2001; Russo et al., 2006). This transport system is involved in symbiosis, since *prsD* mutants of *R. leguminosarum* bv. *viciae* A34 and *R. leguminosarum* bv. *trifolii* TA1 induce nodule formation on their host plants that infect but are unable to fix nitrogen (Finnie et al., 1997; Król and Skorupska, 1997). Krehenbrink and Downie (2008) identified all the extracellular proteins of a closely related strain *R. leguminosarum* bv. *viciae* 3841. Among them were proteins essential for bacterial aggregation and root attachment (RapA2, cadherin-like proteins), motility (two flagellins and flagellar hook protein), EPS maturation (PlyB and PlyA glycanases), uptake of different carbon and nitrogen sources (BraC, dipeptide-binding proteins), and membrane lipoproteins.

On the other hand, diminished amounts of several proteins associated with various transport systems suggested an impairment in the uptake of some nitrogen sources (dipeptides/oligopeptides, branched-chain amino acids) and other compounds (e.g., Fe^3+^ ions) by the *rosR* mutant. This finding is in agreement with our previous results indicating that this mutant utilizes several dipeptides and branched-amino acids less effectively than the wild type (Janczarek et al., 2010). Changes in transport and metabolism can have a negative impact on bacterial symbiotic proficiency since rhizobia have to adapt to specific conditions inside legume nodules (Vercruysee et al., 2011). Although the proteomics results presented in this work correlated well with transcriptomic analysis, one exception were proteins FlaA and FlaB detected in larger amounts in the *rosR* mutant than in the wild-type protein fractions. This emphasizes the notion that proteomic studies allow detection of cell disturbances that cannot be identified using transcriptional analysis. Our particular finding concerning FlaA/B proteins could be explained by improper flagellation of *rosR* mutant cells, where only occasional short flagella were detected (Rachwał et al., 2015). This suggested disturbances in the formation or instability of the flagellar apparatus in the outer membrane, leading to higher amounts of flagellar components in the supernatant, although the expression of genes for these proteins was decreased in the *rosR* mutant. Motility is a rhizobial trait that plays an important role in their competitiveness and survival in various habitats. Fujishige et al. (2006) reported that mutations affecting flagellum formation delay legume nodulation.

Furthermore, reduced amounts of some membrane-associated proteins, such as BamA engaged in membrane protein assembly, may also influence bacterial envelope properties. We observed that the *rosR* mutant was more hydrophobic, with a significantly higher permeability of the outer membrane, than the wild type. Moreover, the size, topography, and surface properties of the mutant cells were different from those of the wild-type cells (Figures 4–6). Our results are similar to those obtained by Dong et al. (2011) for *ctpA* mutant of *R. leguminosarum* bv*. viciae* 3841, which did not synthesize a protease involved in cell envelope functioning. The size and shape of mutant cells were affected, with reduced roughness and adhesion in comparison with wild-type cells. Also, mutations in other rhizobial genes (e.g., *S. meliloti tolC* and *R. leguminosarum* bv. *viciae chvG*) affected envelope integrity (Cosme et al., 2008; Vanderlinde and Yost, 2012). Araujo et al. (1994) characterized *hyd-1::Tn5* mutant of *R. etli* CE3 which was highly hydrophobic and formed domed colonies, similarly to the *rosR* mutant. The growth of the mutant in the rhizosphere was significantly impaired, with reduced nodulation competitiveness, indicating a relationship between cell-surface hydrophobicity and nodulation ability. An intact outer membrane, the permeability barrier that protects the bacterium against harmful compounds while allowing the influx of nutrients, is essential for the proper functioning of a bacterial cell. Hence, the structure and properties of the cell envelope are critical for rhizobial survival under both free-living and symbiotic conditions.

Here, we reported that the observed changes in protein profiles and cell-surface properties caused by the *rosR* mutation significantly affected *R. leguminosarum* bv. *trifolii* symbiosis with the tested clover species (Tables 2, 3). The wild-type strain Rt24.2 was able to nodulate all three tested plant species (*T. pratense, T. repens*, and *T. resupinatum*), but with different effectiveness. *T. pratense* proved to be the best host plant for this strain. Melino et al. (2012) tested *T. subterraneum, T. purpureum*, and *T. polymorphum* and similarly observed that particular *R. leguminosarum* bv. *trifolii* strains were characterized by different symbiotic effectiveness on various *Trifolium* species. Therefore, we analyzed other symbiotic parameters of Rt2472 and Rt24.2 using *T. pratense*. The effectiveness of host root infection by the *rosR* mutant was significantly reduced, and the induced nodules were not adequately occupied by this bacterium (Figures 7–9). Ultrastructural analysis of these nodules revealed disturbances in the IT structure, bacterial release from the ITs, bacteroid differentiation, and H_2_O_2_ accumulation (Figure 10). Proper IT growth and structure are crucial for the establishment of an effective symbiosis. Aberrant ITs in *rosR* mutant nodules were wide, branched, and surrounded by abnormally thick walls, encrusted with additional material, likely calose and/or phenols. Similar IT walls have been described for EPS-, LPS-, and heme-deficient mutants of *R. leguminosarum* bvs. *trifolii* and *viciae* and *S. meliloti* (Dazzo et al., 1991; Dickstein et al., 1991; Cheng and Walker, 1998; Laus et al., 2004; Janczarek et al., 2015a). However, since the *rosR* mutant produces EPS but in diminished amounts in comparison with the wild-type strain (Janczarek et al., 2010), IT structure abnormalities observed in mutant nodules are most probably associated with changes in cell-surface properties and protein profiles. Decreased EPS synthesis is an important but not prevailing cause of this symbiotic defect. Moreover, we hypothesize that disturbances in *rosR* mutant endocytosis are a consequence of inappropriate plant cell cytoskeleton arrangement, caused by alterations in bacterial surface properties. Actin microfilaments associated with unwalled membrane-surrounded droplets are involved in the release of bacteria and the separation of symbiosomes from the IT (Dawidson and Newcomb, 2001). Endocytosis also requires a close interaction between rhizobial cells and the surface of plant cell plasma membrane (Robertson et al., 1985; Rathbun et al., 2002; Bolaños et al., 2004). Thus, the swelling of IT tip and membrane proliferation are most probably caused by improper interactions between the mutant surface and the membrane surrounding the infection droplets.

Furthermore, differentiation of *rosR* mutant bacteroids in the nodules was also strongly impaired (Figure 10). The metabolic exchange between symbiotic partners is essential for proper bacteroid differentiation and productive nitrogen fixation. During the differentiation process, bacteria undergo large-scale physiological and metabolic changes in comparison with their free-living counterparts. As reported by Karunakaran et al. (2009) for *R. leguminosarum* bv. *viciae*, bacteroid metabolism is strongly modulated and an induction of dicarboxylate transport, gluconeogenesis, and alanine synthesis, and repression of sugar utilization is observed. In this bacterium, a mutation of *ptsP* gene encoding phosphotransferase component of the PTS^Ntr^ system that regulates ATP-dependent transporters caused a pleiotropic phenotype similar to that of the *rosR* mutant (the *ptsP* mutant formed dry colonies and grew poorly on organic nitrogen) (Prell et al., 2012). It has been established that legumes regulate bacteroid development and persistence via the supply of branched-chain amino acids. Effective N_2_ fixation by *R. leguminosarum* bv. *viciae* bacteroids requires either one of two broad-specificity amino acid ABC transporters (Aap and Bra) (Prell et al., 2009). Hence, an altered metabolism of the *rosR* mutant is probably one of the important causes of the observed impairment of its symbiotic interaction with the host plant.

Finally, *rosR* mutant nodules differed from wild-type nodules with respect to H_2_O_2_ accumulation (Figure 10). H_2_O_2_ plays a crucial role during different stages of legume–rhizobia symbiosis (i.e., host root infection, IT development, and nitrogen fixation) (Santos et al., 2000; Hérouart et al., 2002; Jamet et al., 2007; Kopcińska, 2009). This compound was detected on mutant bacteroid surface and in the peribacteroid membrane surrounding the symbiosomes, suggesting a reduced ability of the mutant to respond to reactive oxygen species of plant origin. One possible explanation for the impaired adaptation of this strain to conditions inside the host plant cell may be reduction of catalase/hydroperoxidase HPI levels in its periplasmic space. We previously established that the *rosR* mutant was significantly more sensitive to H_2_O_2_ than the wild-type strain *ex planta*, and this stress factor impaired mutant growth and adaptation ability (Jaszek et al., 2014).

In conclusion, the data presented in this study underscore the important role of *rosR* in environmental adaptation in both, free-living stage and during symbiotic interactions with a host plant. The mutation in *rosR* impacted several bacterial characteristics, including size, morphology, envelope properties, protein profiles, and symbiosis with clover. RosR plays an essential role in the *R. leguminosarum* bv. *trifolii* lifestyle and further research should focus on investigating its potential as a target to enhance rhizobium-plant symbiotic interactions.

## Author contributions

KR prepared protein fractions of the rhizobial strains and performed their separation in 1D electrophoresis, carried out AFM analyses, membrane permeability assay, plant experiments, GUS detection in nodules, and participated in protein data analysis from MS; AB conducted protein separations in 2D and western blotting; JK performed electron microscope imaging, discussed the plant data, and participated in writting of the manuscript; MK conducted bacterial hydrophobicity assay, MT participated in protein data analysis from MS; MJ conceived and designed the experiments, participated in isolation of protein fractions, analyzed protein data from MS and other data, discussed the results, wrote the manuscript, and improved the revised version of the manuscript. All authors read and approved the final version of the manuscript.

### Conflict of interest statement

The authors declare that the research was conducted in the absence of any commercial or financial relationships that could be construed as a potential conflict of interest.
